# The burden of psychological distress and unhealthy dietary behaviours among 222,401 school-going adolescents from 61 countries

**DOI:** 10.1038/s41598-023-49500-8

**Published:** 2023-12-11

**Authors:** Md Shajedur Rahman Shawon, Rashawan Raziur Rouf, Esrat Jahan, Fariha Binte Hossain, Sultan Mahmood, Rajat Das Gupta, Md Irteja Islam, Gulam Muhammed Al Kibria, Shariful Islam

**Affiliations:** 1 Centre for Big Data Research in Health, University of New South Wales, Level 2, AGSM Building (G27), Sydney, NSW 2052 Australia; 2 Shaheed Suhrawardy Medical College, Sher-E-Bangla Nagar, Dhaka, Bangladesh; 3 Department of Health Systems and Populations, Macquarie University, Sydney, Australia; 4 School of Population Health, University of New South Wales, Sydney, Australia; 5 University of Western Australia, Perth, WA Australia; 6 Arnold School of Public Health, University of South Carolina, Columbia, USA; 7 School of Public Health, University of Sydney, Sydney, Australia; 8 Johns Hopkins Bloomberg School of Public Health, Baltimore, USA; 9 Institute for Physical Activity and Nutrition (IPAN), Faculty of Health, Deakin University, Perth, Australia

**Keywords:** Human behaviour, Psychology, Health care

## Abstract

We aimed to calculate the sex-specific prevalence of psychological distress and unhealthy eating habits among adolescents across countries and regions, and to explore their potential associations. We used data from the Global School-Based Health Survey (GSHS) for 61 countries. Psychological distress was defined based on the existence of ≥ 2 factors from the following: loneliness, anxiety, suicide ideation, suicide planning, and suicide attempt. Four unhealthy dietary behaviours were examined: inadequate fruit intake, inadequate vegetable intake, daily consumption of soft drinks, and weekly fast-food consumption. We used random-effects meta-analysis to estimate the overall and regional pooled prevalence. Mixed-effect multilevel logistic regressions were used to estimate adjusted odds ratios (aORs) of unhealthy dietary behaviours in relation to psychological distress. Among 222,401 school-going adolescents (53.3% girls), the prevalence of psychological distress was 17.9%, with girls reporting higher than boys (20.8% vs. 14.9%). Adolescents in the African region reported the highest prevalence (22.5%), while those in the South-East Asia region reported the lowest (11.3%). The prevalence of inadequate fruit intake, inadequate vegetable intake, daily soft drink consumption, and weekly fast-food consumption was 37.0%, 28.5%, 50.0%, and 57.4% respectively. Psychological distress was associated with inadequate fruit intake (pooled aOR = 1.19, 95% CI 1.17–1.23), inadequate vegetable intake (pooled OR = 1.19, 1.16–1.22), daily consumption of soft drinks (pooled aOR = 1.14, 1.12–1.17), and weekly consumption of fast food (pooled aOR = 1.12, 1.09–1.15). Our findings indicate a substantial variance in the burden of psychological distress and unhealthy dietary behaviours across different regions. Adolescents experiencing psychological distress were more likely to have unhealthy dietary habits.

## Introduction

The World Health Organisation (WHO) reports that mental health disorders account for approximately 13% of the disease burden in adolescents worldwide^1,2^. These disorders negatively impact physical health, quality of life, academic performance, and economic productivity in adolescence and beyond^3,4^. As a result, adolescent mental health has been recognised as a global health priority^1^. High levels of psychological distress, often manifesting as nonspecific symptoms of stress, anxiety, and depression^5,6^, are indicative of mental health disorders among adolescents^6^. If not detected and treated, psychological distress often escalates to more severe mental health disorders such as personality disorders, major depressive disorder, and suicide^5^. Psychological distress in adolescents has been suggested to be associated with other health risk behaviours, including unhealthy diet^7^, physical inactivity^8^, tobacco use^9^, and harmful alcohol consumption^10^. These health-risk behaviours subsequently amplify the future risk of developing obesity, diabetes, and cardiovascular disease in adulthood^11,12^.

Globally, a considerable number of adolescents exhibit unhealthy or suboptimal dietary habits like insufficient fruit and vegetable intake, consumption of carbonated soft drink, and consumption of fast-food^13–16^. Dietary behaviours during adolescence can be influenced by a variety of factors, including individual or intrapersonal factors (e.g., psychological, biological); social environmental or interpersonal factors (e.g., relationship with family and peers); physical environmental or community settings (e.g., school settings, availability and accessibility to fast food outlets and convenience stores); and macrosystem or societal influences (e.g., social networks, marketing and advertising, and social and cultural norms)^17^. Previous studies have shown that adolescents experiencing psychological distress tend to gravitate towards unhealthy or suboptimal dietary habits^18,19^. Nevertheless, such relationships can be complex to understand and potentially bidirectional in nature^19^.

Although there is a potential relationship between psychological distress and unhealthy dietary practices in adolescents, the current body of evidence has significant limitations due to several issues: (i) a significant proportion of the evidence comes from high-income countries, leaving a gap in data from low- and middle-income countries (LMICs) where around 85–90% of the world's adolescent population resides^19,20^; (ii) it is still unclear whether psychological distress can be linked to various unhealthy dietary behaviours, including fast-food consumption, carbonated soft drink consumption, and low fruit and vegetable intake; (iii) making comparisons across countries or WHO regions is problematic due to differing study populations, variable definitions for psychological distress and unhealthy dietary behaviours, and divergent analytical approaches; and (iv) it remains uncertain if the relationships between psychological distress and unhealthy dietary behaviours can be heterogeneous according to factors such as sex, region, and socioeconomic status. To bridge these research gaps, it is imperative to conduct a comprehensive epidemiological study that includes data from nationally representative samples from various countries in WHO regions. Examining the burden of psychological distress and its associations with unhealthy eating habits would provide valuable information for researchers and policy makers in designing and implementing public health interventions aimed at improving both mental health and dietary practices among adolescents.

The Global School-Based Health Survey (GSHS) offers detailed information on various sociodemographic, psychosocial, lifestyle, and protective factors from nationally representative samples of school-going adolescents from different countries in WHO regions^21^. This enables us to conduct an extensive assessment of psychological distress and unhealthy dietary practices among adolescents, while discerning variations across different countries and WHO regions. Using nationally representative samples from the GSHS in 61 countries, the objectives of this study are to: (i) quantify the prevalence of psychological distress and unhealthy dietary behaviours among school-going adolescents; (ii) compare prevalence estimates by variables such as sex, countries, and WHO regions; and (iii) examine the relationships between psychological distress and unhealthy dietary behaviours, overall, and according to other factors, including sex and WHO regions.

## Methods

### Data sources

We used the most recent GSHS data collected from 61 countries between 2009 and 2018. Surveys from these countries included information relevant to our study, specifically details of variables related to psychological distress and unhealthy dietary habits among school-going adolescents. We excluded GSHS conducted prior to 2009 due to the absence or inconsistent collection of our variables of interest. The GSHS data generally recruited participants aged 11–18 years, and we included all participants regardless of age.

Details about GSHS methodology have been previously elsewhere^21,22^ and are summarised here. GSHS surveys, supported by the World Health Organisation (WHO), the US Centres for Disease Control and Prevention (CDC), aim to assist countries in developing appropriate public health interventions to promote adolescent health. Surveys are conducted in many countries around the world, with the sampling technique remaining consistent between all countries. In summary, a standardised two-stage cluster sampling process was employed to select participating adolescents from each country, a sampling method proportionate to school size was used for school selection, followed by a random selection of classrooms. Then, all the students in the selected classrooms were invited to participate in the survey. During a regular class period, students received a self-administered questionnaire, which was validated and, where necessary, translated into the local language using standard protocols and country-specific examples and phrases to ensure sociocultural adaptation^21^.

Ethics approval for the original surveys was obtained from the appropriate national government body in each participating country, an institutional ethics review committee, or both. Written or verbal informed consent was obtained to participate in the survey from both students and their parents or guardians, regardless of the age of the students. As we used publicly available GSHS data, we did not need to seek separate ethics approval for this study.

### Psychological distress

The GSHS incorporated five questions related to adolescent mental health: feelings of loneliness, anxiety, suicidal ideation, suicide planning, and suicide attempt. We transformed the original responses to these survey questions into binary responses, where 0 indicated 'no' and 1 indicated 'yes.' Following the methodology of previous studies^23^ investigating psychological distress, adolescents who responded 'yes' to two or more of these five variables were categorised as experiencing psychological distress. Additional details on the survey questions and the coding methodology to define psychological distress can be found in Supplementary Table 1.

### Unhealthy dietary behaviours

The GSHS data included information on the consumption of fruits, vegetables, soft drinks, and fast food (see Supplementary Table 1). However, these consumption data were collected in terms of frequency, rather than quantities (e.g., servings). We established the definition of unhealthy dietary behaviours based on these consumption frequencies, following the methodology of a previous global study on dietary behaviour^13^. For fruits and vegetables, we categorised consumption as ‘inadequate’ if adolescents reported eating them less than once per day. Furthermore, we defined daily soft drink consumption and weekly fast-food consumption as unhealthy dietary behaviours. Country-specific examples for each dietary item, with appropriate translations and cultural adaptation, were provided in the questionnaire.

### Statistical analysis

Our analysis was restricted to adolescents who provided valid responses to both psychological distress and unhealthy dietary behaviour variables. We adhered to the data analysis guidelines for GSHS provided by the CDC^21^. To take into account the complex design of the GSHS survey data, we used a sampling weight, a stratification variable, and a primary sampling unit (PSU) variable in the "SVYSET" programme in Stata (version 16.0).

We examined the correlations between variables related to psychological distress and unhealthy dietary behaviours by performing pairwise Pearson correlation tests. We calculated the country-specific prevalence along with 95% confidence intervals (CI) for psychological distress and unhealthy dietary behaviours. Then, we pooled these prevalence estimates by regions and overall using a random-effect meta-analysis in the'metaprop' programme in Stata^24^. We also stratified the prevalence estimates according to sex.

To examine the associations between psychological distress and unhealthy dietary behaviours, we used multilevel mixed-effect logistic regression models with a random intercept to handle common cluster-level random effects within countries^16^. We calculated adjusted odds ratios (OR) with 95% CI, with adjustments for age, sex, WHO region, survey year, hunger (as an indicator for below-average socioeconomic status), close friends, peer support, parental support, being bullied, smoking, physical activity, and being overweight. A detailed account of these variables and their coding can be found in Supplementary Table 1. Any missing or non-applicable values for covariables were categorised separately. We separately analysed the associations for boys and girls to unravel any sex-specific differences in those associations. We also looked for potential effect modification by various factors for the relationships between psychological distress and unhealthy dietary behaviours by comparing ORs across subgroups of these factors and performing likelihood ratio tests to compare models with and without a cross-product interaction term.

Where we presented results as figures, ORs are represented by squares and the corresponding 95% CIs by lines. The area of each square is inversely proportional to the variance of the logarithm of the corresponding estimates, indicating the quantity of statistical information associated with the estimates. Statistical significance was established with a two-tailed p < 0.05.

## Results

In this study, we used GSHS datasets from 61 countries, which included 222,401 school-attending adolescents (53.3% of whom were girls). Table 1 shows the characteristics of the included surveys and the participants. We included countries from five WHO regions: nine from the African region, nine from the Eastern Mediterranean region, 20 from the Region of the Americas, seven from South East Asia and 16 from the Western Pacific region. The sample size varied widely, ranging from 665 in the Cook Islands to 26,222 in Palestine. We included 90.4% of the total sample who provided valid responses to the study's interest variables. The mean age of the total study sample was 14.7 years (SD 1.6), with the mean age ranging from 13.4 years in the Bahamas to 16.4 years in Benin.

**Table 1:** Survey characteristics, by country.

Country	Survey year	n/N	Analysis sample (%)	Boys, n (%)	Girls, n (%)	Mean age (SD)
African region						
Benin	2016	2450/2536	96.6	1328 (54.2)	1122 (45.8)	16.4 (1.6)
Ghana	2012	3375/3632	92.9	1824 (54.0)	1551 (46.0)	16.0 (1.9)
Liberia	2017	2072/2744	75.5	1110 (53.6)	962 (46.4)	16.7 (1.8)
Mauritania	2010	1788/2063	86.7	855 (47.8)	933 (52.2)	14.8 (1.2)
Mauritius	2017	2795/3012	92.8	1290 (46.2)	1505 (53.8)	14.9 (1.4)
Mozambique	2015	1571/1918	81.9	833 (53.0)	738 (47.0)	16.0 (1.8)
Namibia	2013	4101/4531	90.5	1930 (47.1)	2171 (52.9)	15.8 (1.8)
Seychelles	2015	2279/2540	89.7	1038 (45.5)	1241 (54.5)	13.9 (1.5)
Tanzania	2014	3386/3793	89.3	1607 (47.5)	1779 (52.5)	14.3 (1.8)
Eastern Mediterranean region						
Afghanistan	2014	1991/2579	77.2	866 (43.5)	1125 (56.5)	15.2 (1.7)
Bahrain	2016	6807/7141	95.3	3468 (50.9)	3339 (49.1)	14.1 (1.6)
Iraq	2012	1856/2038	91.1	1044 (56.3)	812 (43.8)	14.3 (1.3)
Kuwait	2015	3007/3637	82.7	1376 (45.8)	1631 (54.2)	15.1 (1.6)
Lebanon	2017	5320/5708	93.2	2127 (40.0)	3193 (60.0)	14.9 (1.8)
Morocco	2016	5785/6745	85.8	2999 (51.8)	2786 (48.2)	14.8 (1.9)
Palestine	2010	26,222/29,116	90.1	11,724 (44.7)	14,498 (55.3)	13.9 (1.0)
United Arab Emirates	2016	3224/5849	55.1	1421 (44.1)	1803 (55.9)	15.2 (1.6)
Yemen	2014	2161/2655	81.4	1054 (48.8)	1107 (51.2)	14.9 (1.8)
Region of the Americas						
Anguilla	2016	734/813	90.3	342 (46.6)	392 (53.4)	14.7 (1.3)
Antigua and Barbuda	2009	1135/1266	89.7	522 (46.0)	613 (54.0)	13.9 (0.9)
Bahamas	2013	1229/1357	90.6	552 (44.9)	677 (55.1)	13.4 (1.0)
Belize	2011	1940/2112	91.9	903 (46.5)	1037 (53.5)	13.8 (1.5)
Bolivia	2012	3299/3696	89.3	1685 (51.1)	1614 (48.9)	14.4 (1.1)
British Virgin Islands	2009	1517/1664	91.2	660 (43.5)	857 (56.5)	14.0 (1.5)
Costa Rica	2009	2576/2679	96.2	1245 (48.3)	1331 (51.7)	14.2 (1.1)
Curacao	2015	2542/2765	91.9	1112 (43.7)	1430 (56.3)	15.3 (1.9)
Dominican Republic	2016	1263/1481	85.3	545 (43.2)	718 (56.8)	14.9 (1.5)
El Salvador	2013	1765/1915	92.2	942 (53.4)	823 (46.6)	14.3 (1.0)
Guatemala	2015	3794/4374	86.7	1865 (49.2)	1929 (50.8)	14.4 (1.2)
Honduras	2012	1613/1779	90.7	763 (47.3)	850 (52.7)	13.9 (1.3)
Jamaica	2017	1502/1667	90.1	667 (44.4)	835 (55.6)	15.0 (1.3)
Paraguay	2017	2867/3149	91.0	1362 (47.5)	1505 (52.5)	14.9 (1.5)
Peru	2010	2766/2882	96.0	1344 (48.6)	1422 (51.4)	14.5 (1.0)
Saint Kitts and Nevis	2010	1569/1740	90.2	674 (43.0)	895 (57.0)	14.4 (1.0)
Saint Lucia	2018	1755/1970	89.1	794 (45.2)	961 (54.8)	14.4 (1.7)
Suriname	2016	1970/2126	92.7	953 (48.4)	1017 (51.6)	14.8 (1.7)
Trinidad and Tobago	2017	3459/3869	89.4	1576 (45.6)	1883 (54.4)	14.2 (1.7)
Uruguay	2012	3294/3524	93.5	1525 (46.3)	1769 (53.7)	14.4 (1.0)
South-East Asia Region						
Bangladesh	2014	2787/2989	93.2	1110 (39.8)	1677 (60.2)	14.2 (1.0)
Indonesia	2015	10,602/11,142	95.2	4772 (45.0)	5830 (55.0)	14.1 (1.6)
Maldives	2014	3022/3493	86.5	1235 (40.9)	1787 (59.1)	15.5 (1.5)
Nepal	2015	5957/6529	91.2	2809 (47.2)	3148 (52.8)	14.5 (1.5)
Sri Lanka	2016	3079/3262	94.4	1365 (44.3)	1714 (55.7)	14.7 (1.3)
Thailand	2015	5246/5894	89.0	2133 (40.7)	3113 (59.3)	14.5 (1.7)
Timor-Leste	2015	3115/3704	84.1	1444 (46.4)	1671 (53.6)	15.5 (1.8)
Western Pacific Region						
Brunei Darussalam	2014	2495/2599	96.0	1178 (47.2)	1317 (52.8)	14.6 (1.4)
Cambodia	2013	3631/3806	95.4	1719 (47.3)	1912 (52.7)	15.7 (1.8)
Cook Islands	2015	665/701	94.9	324 (48.7)	341 (51.3)	15.4 (1.4)
Fiji	2016	3260/3705	88.0	1540 (47.2)	1720 (52.8)	15.8 (1.4)
French Polynesia	2015	3064/3216	95.3	1408 (46.0)	1656 (54.0)	15.1 (1.7)
Kiribati	2011	1476/1582	93.3	636 (43.1)	840 (56.9)	14.3 (1.1)
Laos	2015	3554/3683	96.5	1623 (45.7)	1931 (54.3)	15.6 (1.2)
Malaysia	2012	24,694/25,507	96.8	12,237 (49.6)	12,457 (50.4)	14.9 (1.4)
Mongolia	2013	5122/5393	95.0	2381 (46.5)	2741 (53.5)	14.5 (1.7)
Philippines	2015	8188/8761	93.5	3701 (45.2)	4487 (54.8)	14.6 (1.5)
Samoa	2017	1719/1955	87.9	624 (36.3)	1095 (63.7)	15.1 (1.7)
Solomon Islands	2011	1191/1421	83.8	609 (51.1)	582 (48.9)	14.6 (1.3)
Tonga	2017	3021/3333	90.6	1335 (44.2)	1686 (55.8)	14.2 (1.9)
Tuvalu	2013	827/943	87.7	383 (46.3)	444 (53.7)	14.0 (1.5)
Vanuatu	2016	1887/2159	87.4	800 (42.4)	1087 (57.6)	15.0 (1.5)
Wallis and Futuna	2015	1020/1117	91.3	482 (47.3)	538 (52.7)	14.6 (1.8)
Total		222,401/245,959	90.4	103,773 (46.7)	118,628 (53.3)	14.7 (1.6)

n = number of participants who had valid response on psychological distress variables and unhealthy dietary behaviours and included in this analysis.

N = total number of participants included in the GSHS.

**Figure 1:**
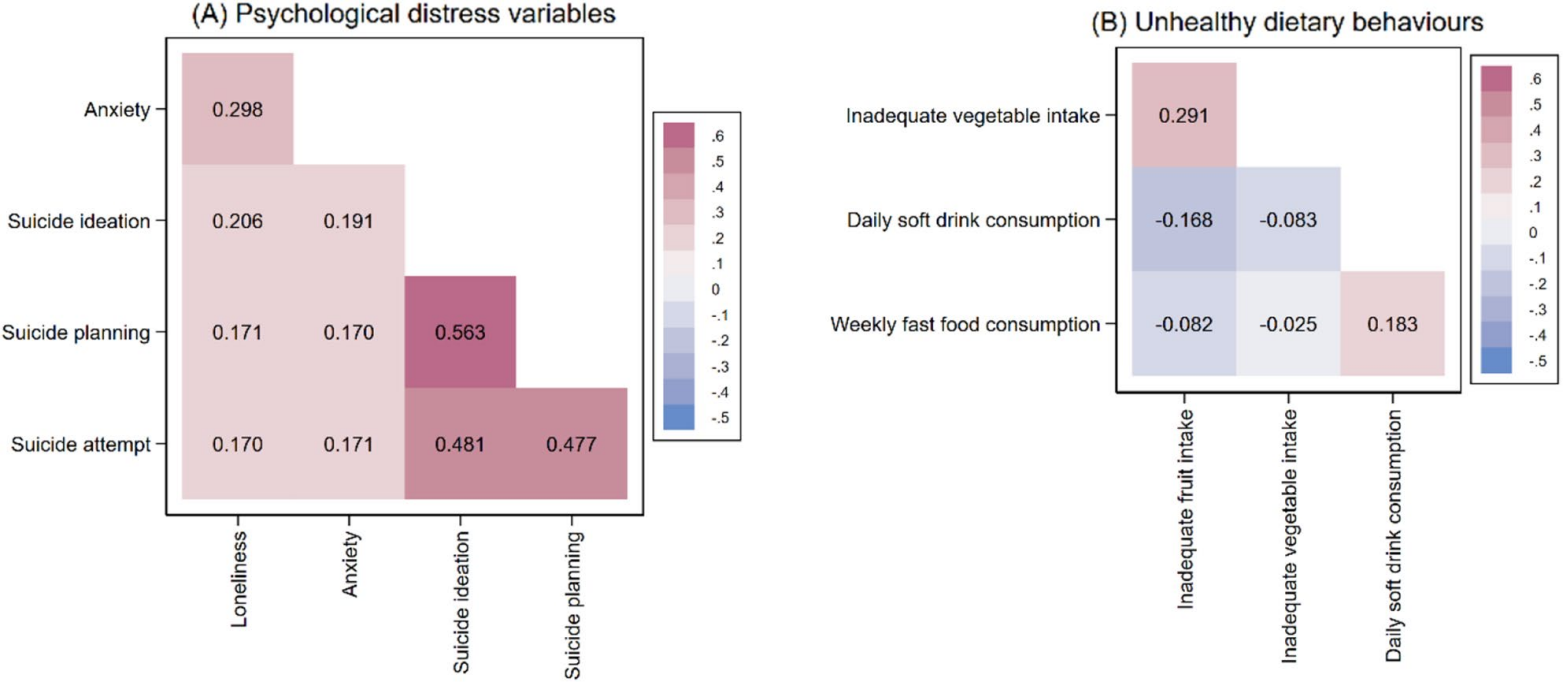
Correlation matrices for (**A**) psychological distress variables and (**B**) unhealthy dietary behaviours. Pairwise Pearson’s correlation coefficients were used to estimate the correlation among the variables.

The correlations between the variables related to psychological distress and unhealthy dietary behaviours are presented in Fig. 1. There were weak to moderate positive correlations among the five variables of psychological distress, suggesting that each represents a slightly different aspect of psychological distress. We noted weak but heterogeneous correlations within the dietary behaviour variables. For example, there was a positive correlation between inadequate vegetable and fruit intake, as well as between weekly fast food consumption and daily soft drink intake. On the contrary, negative correlations were observed between inadequate fruit intake and both daily soft drink consumption and weekly fast-food consumption.

### Prevalence of psychological distress

In general, 17.9% (95% CI 16.2–19.7%) of adolescents experienced psychological distress, with a higher percentage in girls than boys (20.8% vs. 14.9%) (Table 2). This sex difference was consistent across all regions, with the most significant difference observed in the Region of the Americas (23.2% vs. 12.5%). Adolescents from the African region had the highest prevalence of psychological distress (22.5%), while those from the South East Asia region had the lowest prevalence (11.3%). At the country level, Liberia had the highest prevalence (37.1%), while Laos had the lowest prevalence (4.2%). When we separately examined the prevalence of the five underlying factors of psychological distress, we found that the combined prevalence for loneliness, anxiety, suicide ideation, suicide planning, and suicide attempt was 12.1%, 10.7%, 15.3%, 15.0%, and 12.9%, respectively (Supplementary Tables 2–6). Furthermore, girls had a significantly higher prevalence of these factors than boys.

**Table 2:** Country-specific, pooled-regional, and pooled-overall prevalence of psychological distress among school-going adolescents, by sex and overall.

Country	Prevalence (95% CI)*		
	Boys	Girls	Total
African Region			
Benin	20.4 (18.3–22.7)	22.2 (19.8–24.7)	20.9 (19.3–22.5)
Ghana	25.2 (23.2–27.3)	29.9 (27.6–32.2)	27.4 (25.9–29.0)
Liberia	35.6 (32.8–38.5)	38.8 (35.7–41.9)	37.1 (35.0–39.2)
Mauritania	20.4 (17.7–23.2)	17.6 (15.2–20.2)	19.1 (17.3–21.0)
Mauritius	11.3 (9.6–13.2)	19.8 (17.8–21.9)	15.9 (14.6–17.3)
Mozambique	17.6 (15.1–20.4)	22.8 (19.8–26.0)	20.1 (18.1–22.1)
Namibia	28.4 (26.4–30.5)	29.4 (27.5–31.4)	29.0 (27.6–30.4)
Seychelles	16.9 (14.6–19.3)	28.0 (25.5–30.5)	22.6 (20.9–24.4)
Tanzania	10.3 (8.9–11.9)	10.9 (9.5–12.4)	10.6 (9.6–11.7)
Pooled estimates	20.7 (15.3–26.0)	24.3 (18.6–30.1)	22.5 (17.2–27.8)
Eastern Mediterranean region			
Afghanistan	21.1 (18.5–24.0)	32.5 (29.8–35.4)	26.1 (24.2–28.1)
Bahrain	14.2 (13.0–15.4)	21.8 (20.4–23.3)	18.0 (17.1–18.9)
Iraq	17.5 (15.3–20.0)	23.5 (20.6–26.6)	20.1 (18.3–22.0)
Kuwait	18.8 (16.7–20.9)	26.7 (24.5–28.9)	22.8 (21.4–24.4)
Lebanon	11.1 (9.8–12.5)	15.6 (14.4–16.9)	13.6 (12.6–14.5)
Morocco	16.1 (14.8–17.4)	23.8 (22.3–25.5)	19.7 (18.7–20.8)
Palestine	21.0 (20.3–21.8)	24.3 (23.6–25.0)	22.7 (22.2–23.3)
United Arab Emirates	12.7 (11.0–14.6)	19.2 (17.4–21.1)	16.1 (14.8–17.4)
Yemen	19.5 (17.2–22.1)	19.2 (16.9–21.6)	19.3 (17.7–21.1)
Pooled estimates	16.8 (14.1–19.6)	22.9 (20.1–25.7)	19.8 (17.3–22.4)
Region of the Americas			
Anguilla	16.1 (12.4–20.4)	28.6 (24.1–33.3)	22.5 (19.5–25.7)
Antigua and Barbuda	15.3 (12.3–18.7)	25.1 (21.7–28.8)	20.1 (17.8–22.5)
Bahamas	13.2 (10.5–16.3)	23.5 (20.3–26.9)	18.7 (16.6–21.0)
Belize	13.0 (10.8–15.3)	21.0 (18.6–23.6)	17.2 (15.6–19.0)
Bolivia	14.8 (13.2–16.6)	25.1 (23.0–27.3)	19.9 (18.5–21.3)
British Virgin Islands	11.8 (9.5–14.5)	21.7 (19.0–24.6)	17.1 (15.3–19.1)
Costa Rica	6.6 (5.3–8.1)	12.3 (10.6–14.2)	9.4 (8.3–10.6)
Curacao	9.0 (7.4–10.8)	19.9 (17.9–22.1)	14.7 (13.4–16.2)
Dominican Republic	9.0 (6.7–11.7)	24.4 (21.3–27.7)	17.0 (15.0–19.2)
El Salvador	9.3 (7.6–11.4)	18.7 (16.1–21.5)	13.9 (12.4–15.6)
Guatemala	12.0 (10.5–13.5)	20.9 (19.1–22.8)	16.2 (15.1–17.4)
Honduras	13.2 (10.9–15.8)	25.8 (22.9–28.8)	20.0 (18.1–22.1)
Jamaica	18.0 (15.1–21.1)	34.0 (30.8–37.3)	26.4 (24.2–28.7)
Paraguay	9.6 (8.1–11.3)	18.7 (16.8–20.8)	14.3 (13.0–15.6)
Peru	12.6 (10.8–14.5)	27.6 (25.3–30.0)	20.1 (18.6–21.6)
Saint Kitts and Nevis	15.4 (12.8–18.4)	19.2 (16.7–22.0)	17.3 (15.5–19.3)
Saint Lucia	15.4 (12.9–18.1)	31.2 (28.3–34.3)	23.8 (21.8–25.9)
Suriname	12.1 (10.1–14.3)	23.9 (21.3–26.6)	18.2 (16.5–20.0)
Trinidad and Tobago	18.7 (16.8–20.7)	28.4 (26.3–30.5)	23.8 (22.4–25.2)
Uruguay	6.6 (5.4–7.9)	15.7 (14.0–17.4)	11.5 (10.4–12.6)
Pooled estimates	12.5 (10.8–14.1)	23.2 (20.8–25.6)	18.1 (16.1–20.0)
South-East Asia region			
Bangladesh	5.6 (4.3–7.1)	7.7 (6.5–9.1)	6.3 (5.4–7.3)
Indonesia	4.8 (4.2–5.4)	6.0 (5.4–6.7)	5.4 (5.0–5.9)
Maldives	16.9 (14.9–19.1)	21.7 (19.8–23.7)	19.4 (18.0–20.8)
Nepal	12.7 (11.5–14.0)	12.6 (11.5–13.8)	12.7 (11.8–13.5)
Sri Lanka	7.6 (6.3–9.2)	8.7 (7.4–10.1)	8.2 (7.2–9.2)
Thailand	14.0 (12.6–15.6)	14.6 (13.4–15.9)	14.3 (13.4–15.3)
Timor-Leste	14.1 (12.4–16.0)	11.1 (9.6–12.7)	12.6 (11.5–13.8)
Pooled estimates	10.8 (7.1–14.4)	11.7 (8.2–15.3)	11.3 (7.7–14.8)
Western Pacific region			
Brunei Darussalam	7.3 (5.9–8.9)	12.6 (10.9–14.5)	9.9 (8.8–11.1)
Cambodia	7.2 (6.0–8.5)	8.7 (7.5–10.1)	7.9 (7.0–8.8)
Cook Islands	14.2 (10.6–18.5)	18.5 (14.5–23.0)	16.4 (13.7–19.4)
Fiji	13.0 (11.3–14.8)	19.4 (17.5–21.3)	16.4 (15.1–17.7)
French Polynesia	9.9 (8.4–11.6)	22.0 (20.1–24.1)	16.1 (14.8–17.4)
Kiribati	31.8 (28.2–35.5)	34.0 (30.8–37.4)	33.0 (30.6–35.5)
Laos	3.1 (2.3–4.1)	5.4 (4.4–6.5)	4.2 (3.6–4.9)
Malaysia	6.6 (6.1–7.0)	9.7 (9.2–10.2)	8.1 (7.8–8.5)
Mongolia	13.8 (12.4–15.2)	20.6 (19.1–22.2)	17.4 (16.3–18.4)
Philippines	12.0 (10.9–13.1)	18.8 (17.7–20.0)	15.5 (14.7–16.3)
Samoa	22.9 (19.7–26.4)	23.7 (21.3–26.4)	23.3 (21.3–25.4)
Solomon Islands	33.5 (29.8–37.4)	32.3 (28.5–36.3)	33.0 (30.3–35.7)
Tonga	18.0 (16.0–20.1)	18.7 (16.8–20.6)	18.3 (17.0–19.8)
Tuvalu	10.7 (7.8–14.2)	6.8 (4.6–9.5)	8.7 (6.9–10.8)
Vanuatu	23.3 (20.4–26.3)	16.0 (13.9–18.3)	19.5 (17.7–21.4)
Wallis and Futuna	22.2 (18.6–26.2)	32.2 (28.2–36.3)	27.4 (24.6–30.2)
Pooled estimates	15.3 (12.4–18.2)	18.6 (15.1–22.0)	17.1 (13.9–20.2)
Overall estimate†	14.9 (13.3–16.5)	20.8 (18.8–22.8)	17.9 (16.2–19.7)

*Country-specific sampling weights were used to yield country representative estimates.

^†^Random-effect meta-analysis was used to calculate the pooled estimates.

### Prevalence of unhealthy dietary behaviours

Overall, 37% (95% CI 34.7–39.4%) of school-attending adolescents in all regions reported consuming fruits less than once a day, while the figure for vegetable consumption less than once a day was 28.5% (95% CI 26.1–30.8%) (Table 3). We found that 50% (95% CI 46.5–53.6%) and 57.4% (95% CI 54.0–60.8%) of all adolescents reported daily intake of soft drinks and weekly intake of fast food, respectively. Adolescents in the South-East Asia Region had a significantly higher prevalence of inadequate fruit intake (45.3%) compared to other regions, while we did not observe significant differences for inadequate vegetable intake between regions. Notable differences were found in the prevalence of daily soft drink intake (lowest in the South-East Asia region [37.2%] and highest in the region of the Americas [62.7%]). For the weekly intake of fast food, adolescents in the African region had the lowest prevalence (52.3%), and those in the Eastern Mediterranean region had the highest prevalence (63.4%) (Table 3).

**Table 3:** Prevalence of unhealthy dietary behaviours among school-going adolescents, overall and by region.

Region	Prevalence (95% CI)*			
	Inadequate fruit intake	Inadequate vegetable intake	Daily soft drink intake	Weekly fast-food intake
African region				
Boys	37.6 (33.0–42.1)	30.2 (23.7–36.7)	45.2 (39.0–51.5)	51.3 (43.4–59.3)
Girls	35.3 (30.5–40.1)	28.1 (21.9–34.3)	51.0 (45.1–57.0)	53.5 (44.4–62.5)
Total	36.5 (31.9–41.1)	29.3 (23.0–35.7)	47.8 (41.7–53.9)	52.3 (43.9–60.6)
Eastern Mediterranean region				
Boys	37.2 (31.5–43.0)	30.3 (24.7–35.8)	44.4 (36.8–51.9)	66.7 (58.8–74.6)
Girls	35.4 (28.5–42.3)	28.9 (23.6–34.2)	38.5 (30.3–46.7)	59.7 (48.3–71.1)
Total	36.6 (30.4–42.8)	29.7 (24.4–35.1)	41.5 (33.8–49.1)	63.4 (53.8–72.9)
Region of Americas				
Boys	34.5 (31.3–37.6)	28.1 (24.6–31.7)	63.2 (59.8–66.7)	56.4 (53.0–59.9)
Girls	34.8 (30.8–38.8)	28.5 (24.9–32.1)	62.3 (58.2–66.3)	60.3 (56.5–64.0)
Total	34.7 (31.2–38.2)	28.4 (24.8–31.9)	62.7 (59.1–66.4)	58.4 (54.9–61.9)
South-East Asia region				
Boys	45.8 (37.4–54.2)	31.1 (20.2–41.9)	38.6 (30.6–46.6)	57.6 (46.2–69.1)
Girls	44.9 (34.4–55.5)	28.5 (16.2–40.8)	36.0 (27.5–44.5)	58.5 (45.3–71.7)
Total	45.3 (35.9–54.7)	29.8 (18.2–41.4)	37.2 (29.2–45.3)	58.3 (46.1–70.5)
Western Pacific region				
Boys	38.0 (33.4–42.5)	27.5 (24.3–30.8)	45.6 (40.0–51.3)	55.7 (49.0–62.4)
Girls	35.6 (30.6–40.6)	25.6 (22.4–28.8)	46.1 (39.0–53.2)	54.9 (48.0–61.8)
Total	36.8 (32.1–41.5)	26.7 (23.6–29.9)	45.9 (39.7–52.2)	55.3 (48.6–62.0)
All regions				
Boys	37.6 (35.3–39.8)	29.0 (26.7–31.2)	50.3 (46.9–53.8)	57.1 (53.8–60.5)
Girls	36.4 (33.8–38.9)	27.8 (25.3–30.3)	49.8 (46.0–53.6)	57.6 (53.9–61.2)
Total	37.0 (34.7–39.4)	28.5 (26.1–30.8)	50.0 (46.5–53.6)	57.4 (54.0–60.8)

*Country-specific sampling weights were used to yield country representative estimates and random-effect meta-analysis was used to calculate the pooled prevalence estimates.

*Country-specific prevalence estimates are given in supplementary tables S7-S10.

No significant sex differences were observed for the overall prevalence of these unhealthy dietary behaviours. However, considerable sex disparities were observed for the daily intake of soft drinks and the weekly intake of fast foods in some regions. For example, adolescent girls in the African region were more likely to consume soft drinks daily than boys (51.0% vs. 45.2%), whereas more boys in the Eastern Mediterranean region consumed soft drinks daily than girls (44.4% vs. 38.5%) (Table 3). In the Region of Americas, 60.3% of girls had weekly fast-food consumption compared to 56.4% boys; however, the reverse was true in the Eastern Mediterranean region, with 66.7% of boys and 59.7% of girls reporting weekly fast-food consumption.

The country-specific prevalence of these unhealthy dietary behaviours is provided in Supplementary Tables 7–10. For inadequate fruit intake, Vanuatu (23.2%) had the lowest prevalence, and Maldives had the highest prevalence (63.0%). Maldives also had the highest prevalence for inadequate vegetable intake (63.5%), while Sri Lanka had the lowest prevalence (9.5%). Adolescents in Suriname had the highest prevalence of daily soft drink intake (78.9%), and those in Kiribati had the lowest prevalence (21.7%). Almost 4 in 5 adolescents in Thailand had consumed fast food weekly, while only 1 in 5 adolescents in Cambodia reported weekly fast food consumption.

### Associations between psychological distress and unhealthy dietary behaviours

**Figure 2:**
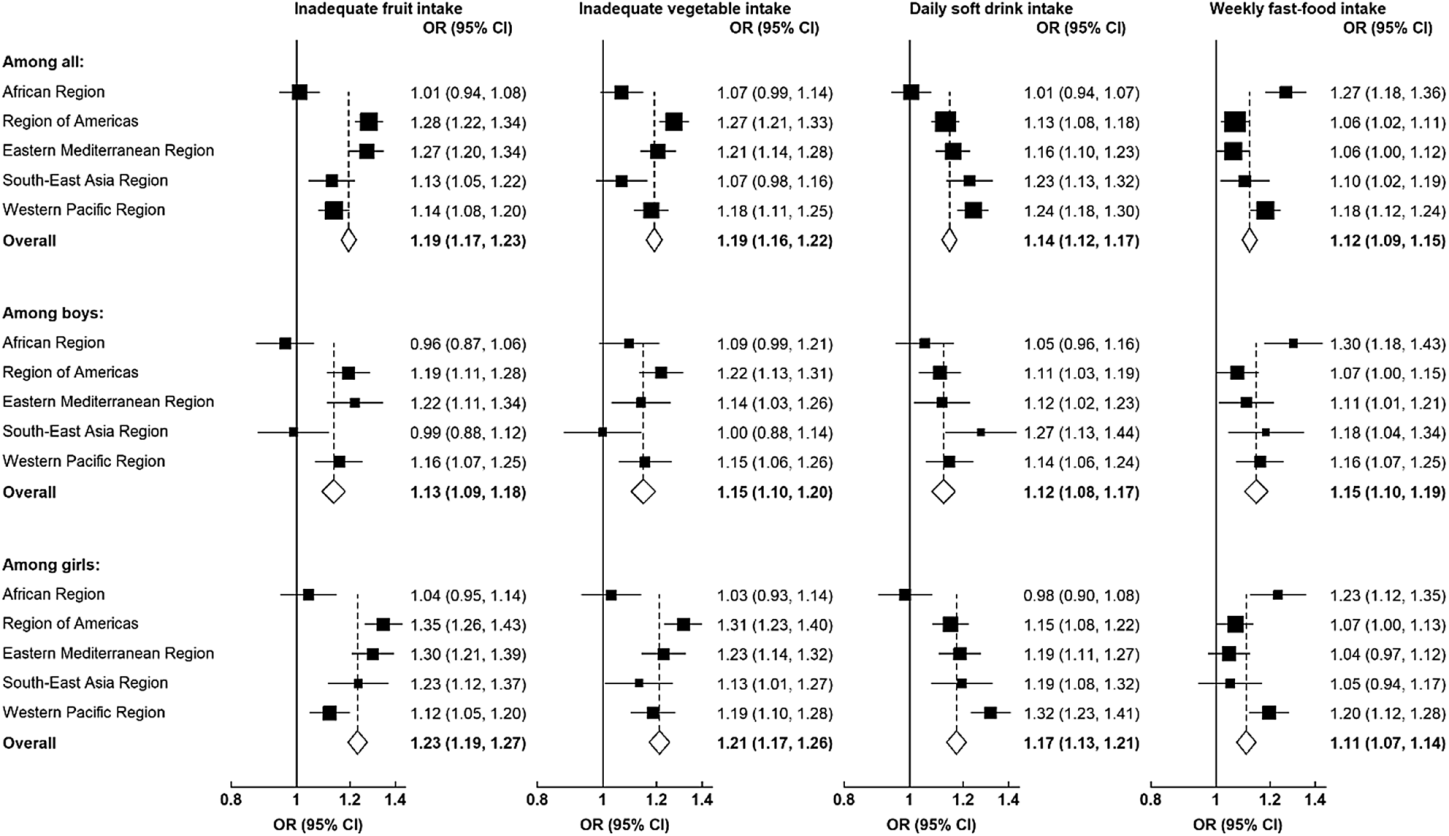
Associations of psychological distress with unhealthy dietary behaviours among adolescents, overall and by region. Multi-level mixed-effect logistic regressions were adjusted for age, sex, low socioeconomic status, survey year, close friend, bullying, parental support, peer support, cigarette smoking, physical activity, and overweight. Odds ratios (ORs) are represented by squares, and their corresponding 95% CIs are represented by lines. The area of each square is inversely proportional to the variance of the logarithm of the corresponding OR estimates, which shows the amount of statistical information involved with the estimates.

Figure 2 shows the relationship between psychological distress and unhealthy dietary behaviours. Overall, there was a significant association between psychological distress and inadequate fruit intake (pooled OR = 1.19, 95% CI 1.17–1.23), inadequate vegetable intake (pooled OR = 1.19, 95% CI 1.16–1.22), daily soft drink consumption (pooled OR = 1.14, 95% CI 1.12–1.17), and weekly fast-food consumption (pooled OR = 1.12, 95% CI 1.09–1.15). We noted considerable variations in region-specific ORs, illustrating heterogeneity in the associations between psychological distress and unhealthy dietary behaviours. For example, the African region alone did not show significant associations between psychological distress and inadequate fruit or vegetable intake, as well as daily soft drink consumption. However, adolescents from the Western Pacific region showed stronger associations between psychological distress and daily soft drink consumption and weekly fast-food consumption, in contrast to other regions.

When examining the associations separately for boys and girls, we found that psychological distress was significantly associated with all four unhealthy dietary behaviours in both sexes (Fig. 2). However, these associations were slightly stronger in girls than in boys when it came to insufficient fruit consumption (pooled OR 1.23 vs. 1.13), insufficient vegetable consumption (pooled OR 1.21 vs. 1.15), and daily soft drink consumption (pooled OR 1.17 vs. 1.12). In particular, psychological distress was only associated with inadequate fruit and vegetable intake among girls in the South East Asia region.

**Figure 3:**
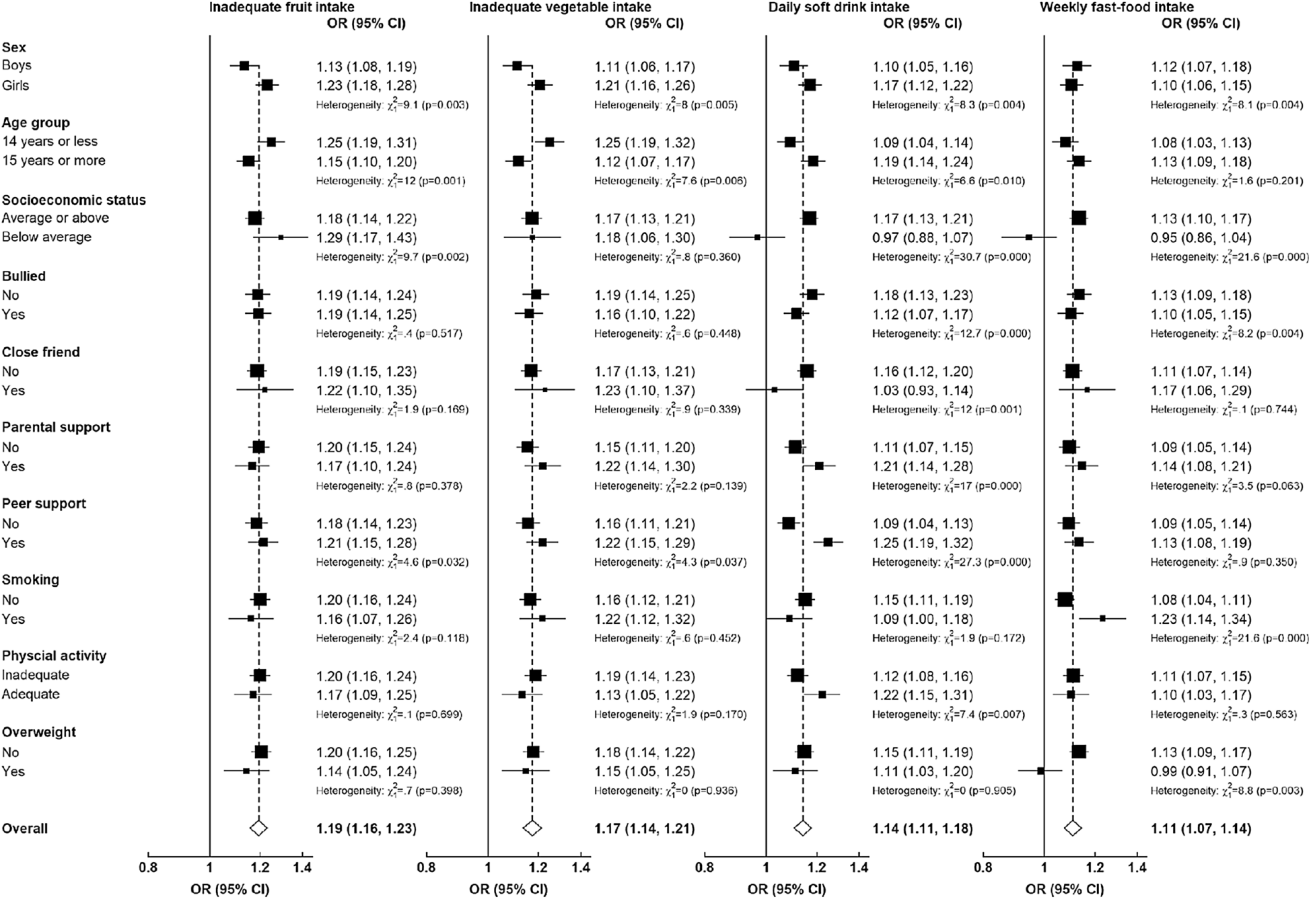
Associations of psychological distress with unhealthy dietary behaviours among adolescents, by levels of other factors. Multi-level mixed-effect logistic regressions were adjusted for age, sex, low socioeconomic status, survey year, region, bullying, close friend, parental support, peer support, smoking, physical activity, and overweight, as appropriate. Heterogeneity between groups was tested by likelihood ratio tests comparing models with and without cross product interaction term.

We also examined the relationships between psychological distress and four unhealthy dietary behaviours in various subgroups of individual characteristics (Fig. 3). We observed significant differences between the sexes in these relationships. In addition, we found variations in these associations based on age group and socioeconomic status. The correlation between psychological distress and daily soft drink consumption showed significant variation based on factors such as bullying, having close friends, parental support, peer support, and physical activity. Significant differences also emerged in the association between psychological distress and weekly fast-food consumption based on levels of bullying, smoking, and being overweight (Fig. 3).

## Discussion

In this large study based on nationally representative samples of school-going adolescents from 61 countries in different WHO regions, we found that more than one in six adolescents had psychological distress. Girls had a higher prevalence of distress than boys. The prevalence of inadequate fruit intake, inadequate vegetable intake, daily consumption of soft drinks and weekly fast food consumption was substantially high. There were substantial variations in the prevalence of psychological distress and unhealthy dietary behaviours across WHO regions. The presence of psychological distress was significantly associated with the four unhealthy dietary behaviours. There was evidence of heterogeneity in the associations observed by factors such as sex, region, and socioeconomic status.

Our study showed a significantly high prevalence of psychological distress among adolescents across all regions, aligning with previous research indicating a high global burden of mental health disorders among this group^1,20,25,26^. However, our results are not directly comparable with these prior studies due to differences in the age distribution of the samples and variable definitions. For example, a recent systematic review reported that 31% of all adolescents aged 10–19 years suffer from common mental health disorders, based on studies using the General Health Questionnaire (GHQ-12)^26^. In contrast, another study drawn from the Global Burden of Disease Study estimated the prevalence of mental health disorders, including depression, anxiety, conduct disorder, attention deficit / hyperactivity disorder, autism spectrum disorders, and eating disorders among those aged 5–17 years of age and reported that the average prevalence of mental health disorders was 6.7%. However, crucially, data on mental health disorders were missing for 124 of the 187 countries in that study^20^. The variation in the prevalence of psychological distress in WHO regions can be due to differences in psychological, cultural, economic, religious, and political stressors^27^. We observed the highest prevalence of psychological distress (22.5%) in the African region. A recent systematic review of adolescent mental health problems in sub-Saharan Africa also reported high rates of depression, anxiety disorders, emotional and behavioural difficulties, post-traumatic stress, and suicidal behaviour^28^. Factors such as the high prevalence of HIV/AIDS, malaria, displacement, human rights violations, and impoverishment could potentially escalate the risk of mental health problems among African adolescents^29^.

According to previous research^1,20,25,26^ our study also indicates that adolescent girls suffer a higher burden of psychological distress than boys. We found similar sex disparities when examining the prevalence of loneliness, anxiety, suicidal ideation, suicide planning, and suicide attempts separately. An earlier analysis of GSHS data found that suicidal behaviour was more prevalent among adolescent girls (26.2%) than among boys (23.0%)^24^. Another study involving adolescents from 73 OECD countries found that girls exhibited more severe forms of psychological distress than boys^30^. This sex disparity in psychological distress could be attributed to the higher rates of internalising problems, domestic violence, and sexual abuse of girls^30,31^. In addition, the stress of juggling multiple gender norms (such as participating in academic and economic activities while preserving feminine identity and appearance) may also trigger more anxiety in girls than in boys^32^.

Our study also reveals that a significantly high proportion of adolescents are engaged in unhealthy dietary habits. Although we did not find significant sex differences in these unhealthy dietary behaviours, there were significant variations between WHO regions. The GSHS does not provide specific quantities for fruit and vegetable intake, so we defined inadequate intake based on consumption frequency, using cutoffs from a previous global study on adolescent dietary behaviour^13^. According to the WHO, the minimum recommended daily intake of fruits and vegetables is 400 g^33^. Given the low consumption frequencies in our study, it is likely that the actual intake fell below this minimum recommendation. Furthermore, our findings emphasise that the percentage of adolescents consuming inadequate amounts of fruit was higher than those with inadequate vegetable consumption in all regions. The reasons for this deficiency could include supply problems, financial constraints, increased prices due to urbanisation, and changes in food shopping trends in many countries^34^. We also found high rates of fast food and soft drink consumption among adolescents in all regions, reflecting the widespread availability of street food and fast food outlets due to rapid changes in food culture and McDonaldization of the food environment^35^.

Our study demonstrates that adolescents experiencing psychological distress are more likely to have unhealthy dietary behaviours such as low fruit and vegetable intake and high consumption of soft drinks and fast food. Previous studies investigating the links between mental health issues and diet in adolescents have found that 'Westernised' eating patterns are associated with psychological distress^19,36–38^. A recent systematic review found consistent associations between mental health disorders like depression, anxiety, and low mood and unhealthy dietary patterns in children and adolescents^19^. A study among Chinese adolescents identified a positive link between soft drink and sweet food consumption and the risk of suicidal behaviour^39^. Understanding the relationships between mental health problems and diet among adolescents is highly complex, as observed associations may be bidirectional and potentially affected by confounding and reporting biases^18,19^. While one could hypothesise that psychological distress promotes unhealthy dietary habits among adolescents, others could argue that an unhealthy diet could increase the risk of developing psychological distress^16^. Although we cannot establish causality for the observed associations, it is conceivable that low mood might lead to increased consumption of unhealthy foods such as fast foods and chocolate and a decrease in the intake of healthier foods. Palatable food may offer comfort, especially to those who are high emotional eaters, while they are eating^40^. Future prospective studies are needed to understand the causal mechanisms underlying the association between psychological distress and unhealthy dietary behaviours among adolescents. Given the significant regional variations observed in our study, further investigation is also needed to understand how cultural, socioeconomic, and environmental factors influence these patterns. These inquiries will be crucial for developing targeted interventions and public health strategies to address these interconnected issues in diverse adolescent populations worldwide.

Our study has several strengths. First, we included a large number of school-going adolescents from nationally representative samples from 61 countries in five WHO regions. This would facilitate the generalisation of our prevalence estimates to the adolescent population of relevant countries and WHO regions. Second, the GSHS used standardised methodologies for participant recruitment, questionnaire creation, and data collection in all participating countries. In addition, we used consistent definitions for both exposure and outcome variables. As a result, the cross-country and cross-regional comparisons of our study are likely to be more valid. Thirdly, we used mixed-effect multilevel logistic regression models with adjustments for a broad range of covariates while examining the relationships between psychological distress and unhealthy dietary behaviours. Furthermore, we investigated the possibility of further modification of the effect by various factors such as age, sex, and socioeconomic status.

Despite these strengths, there are several constraints to consider in our study. We determined psychological distress in adolescents based on self-reported data on five mental health aspects, as outlined in previous studies^23^. We recognise that the interpretation of a multifaceted and complex phenomenon like psychological distress can remain incomplete and may differ from many previous studies using other methodologies. Unhealthy dietary behaviours were identified based on self-reported food frequency from the last 30 days, rather than the amount consumed, which is less accurate than 24-h recall studies^16^. Furthermore, self-reported measures could introduce bias; for example, discussing mental health problems may be considered taboo in certain cultures and countries. Our definition of 'inadequate' as less than once per day is indeed a conservative benchmark. It does not align with higher standards, such as the UK’s 5 + a day target, and this limitation in our criteria should be considered when interpreting our findings. The data incorporated in this study span GSHS surveys conducted from 2009 to 2018. Given the increasing prevalence of mental health disorders among adolescents in recent years, our estimates of psychological distress should be interpreted with caution. GSHS data are based on responses from school-attending adolescents, who could differ in several ways from their out-of-school counterparts. According to World Bank data, rate of secondary school enrolment in LMICs is 63%^41^. The variation in data collection years (2009–2018) in our study also introduces a limitation, as it may affect the accuracy of comparing current trends in psychological distress and unhealthy habits across countries and regions. Finally, the cross-sectional nature of our data prevents us from establishing a chronological sequence of observed associations between psychological distress and unhealthy eating habits. Although we considered multiple covariates in our analyses, there could still be residual confounding due to unmeasured variables or problems with the measurements in the adjusted variables.

In conclusion, our study highlighted the high burden of psychological distress and unhealthy dietary behaviours among adolescents in all WHO regions. Adolescents experiencing psychological distress were more likely to have unhealthy dietary behaviours. There were important differences in the prevalence of psychological distress and unhealthy diets in regions, and the associations observed for them were consistently strong in all regions except the African region. These findings can inform public health initiatives aimed at improving adolescent mental health and eating practices, rationalise resource allocation, and guide policymaking in various regions.

### Supplementary Information

Supplementary Tables.

## Data Availability

Global School-based Student Health Survey (GSHS) datasets used in this study are publicly available at this link: https://extranet.who.int/ncdsmicrodata/index.php/home.
