# Supplementary material for: The burden of psychological distress and unhealthy dietary behaviours among 222,401 school-going adolescents from 61 countries
Source: Sci Rep. 2023 Dec 11;13:21894. doi: 10.1038/s41598-023-49500-8 (PMC10713667; doi:10.1038/s41598-023-49500-8)
Supplement: Supplementary file 1 — Supplementary Tables. [file 41598_2023_49500_MOESM1_ESM.docx]

**The burden of psychological distress and unhealthy dietary behaviours among 222,401 school-going adolescents from 61 countries**

**Supplementary materials**

**Supplementary Table 1: Variable definitions**

| **Variables** | **Survey question** | **Response options and recording** |
| --- | --- | --- |
| **Psychological distress variables** | | |
| Anxiety | During the past 12 months, how often have you been so worried about something that you could not sleep at night? | No = “never” or “rarely” or “sometimes”  Yes = “most of the times” or “always” |
| Loneliness | During the past 12 months, how often have you felt lonely? | No = “never” or “rarely” or “sometimes”  Yes = “most of the times” or “always” |
| Suicide ideation | During the past 12 months, did you ever seriously consider attempting suicide? | No  Yes |
| Suicide planning | During the past 12 months, did you make a plan about how you would attempt suicide? | No  Yes |
| Suicide attempt | During the past 12 months, how many times did you actually attempt suicide? | No = 0 time  Yes = 1, 2 or 3, 4 or 5, 6 or more times |
| Psychological distress |  | No = Presence of less than two out of five psychological variables  Yes = Presence of two or more out of five psychological variables |
| **Unhealthy dietary behaviours** | | |
| Fruit consumption | During the past 30 days, how many times per day did you **usually** eat fruit, such as COUNTRY SPECIFIC EXAMPLES? | Inadequate = I did not eat fruit during the past 30 days or Less than one time per day  Adequate = 1 time per day, 2 times per day,  3 times per day, 4 times per day, or 5 or more times per day |
| Vegetable consumption | During the past 30 days, how many times per day did you **usually** eat fruit, such as COUNTRY SPECIFIC EXAMPLES? | Inadequate = I did not eat fruit during the past 30 days or Less than one time per day  Adequate = 1 time per day, 2 times per day,  3 times per day, 4 times per day, or 5 or more times per day |
| Soft drink consumption | During the past 30 days, how many times per day did you **usually** drink carbonated soft drinks, such as COUNTRY SPECIFIC EXAMPLES? (Do **not** include diet soft drinks.) | Daily = 1 time per day, 2 times per day,  3 times per day, 4 times per day, or  5 or more times per day  Less than daily = I did not drink carbonated soft drinks during the past 30 days or Less than 1 time per day |
| Fast food consumption | During the past 7 days, on how many days did you eat food from a fast food restaurant, such as COUNTRY SPECIFIC EXAMPLES? | Less than weekly = 0 days  Weekly = 1 day, 2 days, 3 days, 4 days, 5 days,  6 days, or 7 days |
|  |  |  |
| **Covariates** |  |  |
| Age | How old are you? |  |
| Sex | What is your sex? |  |
| Socioeconomic status (huger as proxy) | During the past 30 days, how often did you go hungry because there was not enough food in your home? | Average = “never” or “rarely” or “sometimes”  Below average = “most of the times” or “always” |
| Bullied | During the past 30 days, on how many days were you bullied? | No = 0 days  Yes = “1 or 2 days” or “3 to 5 days” or “6 to 9 days” or “10 to 19 days” or “20 to 29 days” or “All 30 days” |
| Parental support | During the past 30 days, how often did your parents or guardians understand your problems and worries? | No = “never” or “rarely” or “sometimes”  Yes = “most of the times” or “always” |
| Peer support | During the past 30 days, how often were most of the students in your school kind and helpful? | No = “never” or “rarely” or “sometimes”  Yes = “most of the times” or “always” |
| Close friend | How many close friends do you have? | No = 0  Yes = 1, 2, 3 or more |
| Smoking | During the past 30 days, on how many days did you smoke cigarettes? | No = “0 days”  Yes = “1 or 2 days” or “3 to 5 days” or ‘”6 to 9 days” or “10 to 19 days” or “20 to 29 days” or “All 30 days” |
| Physical activity | During the past 7 days, on how many days were you physically active for a total of at least 60 minutes per day? ADD UP ALL THE TIME YOU SPENT IN ANY KIND OF PHYSICAL ACTIVITY EACH DAY. | No = “0 days”  Yes = “1 day” or “2 days” or “3 days” or “4 days” or “5 days” or “6 days” or “7 days” |
| Overweight |  | Body mass index (BMI) is >+1SD from the median for age and sex, according to the WHO Growth Reference Data |

More details about GSHS questionnaires can be found at: <https://www.who.int/ncds/surveillance/gshs/methodology/en/>

| **Supplementary Table 2: Country-specific, pooled-regional, and pooled-overall prevalence of loneliness among school-going adolescents, by sex and overall** | | | |
| --- | --- | --- | --- |
| **Country** | **Prevalence (95% CI)*** | | |
|  | **Boys** | **Girls** | **Total** |
| **African Region** | |  |  |
| Benin | 13.7 (11.9-15.7) | 14.3 (12.3-16.5) | 13.9 (12.5-15.3) |
| Ghana | 14.3 (12.7-15.9) | 15.2 (13.5-17.1) | 14.7 (13.5-15.9) |
| Liberia | 11.3 (9.5-13.3) | 14.2 (12.1-16.6) | 12.6 (11.2-14.2) |
| Mauritania | 15.0 (12.6-17.5) | 14.1 (12.0-16.6) | 14.6 (13.0-16.3) |
| Mauritius | 9.0 (7.5-10.7) | 11.8 (10.2-13.5) | 10.5 (9.4-11.7) |
| Mozambique | 8.6 (6.8-10.8) | 10.8 (8.7-13.3) | 9.7 (8.3-11.2) |
| Namibia | 12.5 (11.0-14.0) | 17.1 (15.5-18.7) | 14.9 (13.8-16.1) |
| Seychelles | 6.4 (5.0-8.0) | 15.2 (13.3-17.4) | 10.9 (9.7-12.3) |
| Tanzania | 6.3 (5.1-7.6) | 6.7 (5.6-8.0) | 6.5 (5.7-7.4) |
| *Pooled estimates* | *10.7 (8.5-13.0)* | *13.3 (10.7-15.9)* | *12.0 (9.8-14.2)* |
|  |  |  |  |
| **Eastern Mediterranean Region** | | | |
| Afghanistan | 23.0 (20.2-25.9) | 41.5 (38.6-44.5) | 31.1 (29.1-33.2) |
| Bahrain | 12.1 (11.1-13.3) | 19.6 (18.3-21.0) | 15.9 (15.0-16.8) |
| Iraq | 13.4 (11.4-15.6) | 20.2 (17.5-23.1) | 16.4 (14.7-18.1) |
| Kuwait | 14.2 (12.4-16.2) | 22.2 (20.2-24.3) | 18.4 (17.0-19.8) |
| Lebanon | 7.3 (6.2-8.5) | 15.0 (13.7-16.3) | 11.4 (10.6-12.3) |
| Morocco | 13.8 (12.6-15.1) | 24.8 (23.2-26.4) | 19.0 (18.0-20.0) |
| Palestine | 11.0 (10.5-11.6) | 16.5 (15.9-17.1) | 13.9 (13.4-14.3) |
| United Arab Emirates | 10.6 (9.0-12.3) | 15.9 (14.3-17.7) | 13.3 (12.2-14.6) |
| Yemen | 14.1 (12.1-16.4) | 20.8 (18.4-23.3) | 17.2 (15.6-18.8) |
| *Pooled estimates* | *13.1 (11.2-15.0)* | *21.7 (18.2-25.2)* | *17.3 (14.9-19.7)* |
|  |  |  |  |
| **Region of the Americas** | | |  |
| Anguilla | 9.4 (6.5-13.0) | 18.4 (14.7-22.6) | 13.9 (11.5-16.6) |
| Antigua and Barbuda | 10.2 (7.7-13.1) | 15.8 (13.0-19.0) | 13.0 (11.1-15.0) |
| Bahamas | 7.6 (5.5-10.1) | 13.4 (11.0-16.2) | 10.7 (9.1-12.6) |
| Belize | 9.3 (7.5-11.4) | 15.7 (13.6-18.1) | 12.7 (11.3-14.3) |
| Bolivia | 6.9 (5.8-8.3) | 14.3 (12.6-16.1) | 10.5 (9.5-11.6) |
| British Virgin Islands | 8.3 (6.3-10.7) | 12.4 (10.2-14.8) | 10.5 (9.0-12.1) |
| Costa Rica | 3.9 (2.9-5.1) | 9.2 (7.7-10.8) | 6.5 (5.6-7.5) |
| Curacao | 10.3 (8.5-12.2) | 20.6 (18.6-22.8) | 15.7 (14.3-17.2) |
| Dominican Republic | 7.3 (5.3-9.9) | 18.9 (16.1-22.0) | 13.4 (11.6-15.4) |
| El Salvador | 4.6 (3.3-6.1) | 12.9 (10.7-15.4) | 8.6 (7.3-10.0) |
| Guatemala | 4.6 (3.7-5.7) | 11.8 (10.4-13.3) | 8.1 (7.2-9.0) |
| Honduras | 6.7 (5.0-8.7) | 14.2 (12.0-16.8) | 10.8 (9.3-12.4) |
| Jamaica | 12.9 (10.4-15.7) | 23.7 (20.9-26.7) | 18.5 (16.6-20.6) |
| Paraguay | 8.6 (7.2-10.2) | 13.0 (11.4-14.8) | 10.8 (9.7-12.0) |
| Peru | 6.8 (5.6-8.3) | 14.0 (12.2-15.9) | 10.4 (9.3-11.6) |
| Saint Kitts and Nevis | 10.2 (8.1-12.8) | 13.0 (10.8-15.3) | 11.6 (10.1-13.3) |
| Saint Lucia | 14.1 (11.8-16.7) | 23.0 (20.4-25.8) | 18.9 (17.1-20.8) |
| Suriname | 13.9 (11.7-16.2) | 19.8 (17.4-22.3) | 16.9 (15.3-18.6) |
| Trinidad and Tobago | 12.2 (10.6-13.9) | 16.3 (14.6-18.0) | 14.3 (13.2-15.6) |
| Uruguay | 3.8 (2.9-4.9) | 9.8 (8.5-11.3) | 7.1 (6.2-8.0) |
| *Pooled estimates* | *8.5 (7.1-9.9)* | *15.4 (13.7-17.1)* | *12.1 (10.6-13.6)* |
|  |  |  |  |
| **South-East Asia Region** | | |  |
| Bangladesh | 10.6 (8.9-12.6) | 9.2 (7.8-10.7) | 10.1 (9.0-11.3) |
| Indonesia | 4.9 (4.3-5.6) | 6.6 (6.0-7.3) | 5.8 (5.4-6.3) |
| Maldives | 12.5 (10.7-14.4) | 18.6 (16.9-20.5) | 15.6 (14.3-16.9) |
| Nepal | 6.1 (5.2-7.0) | 5.5 (4.7-6.3) | 5.8 (5.2-6.4) |
| Sri Lanka | 7.8 (6.5-9.4) | 9.4 (8.1-10.9) | 8.6 (7.6-9.7) |
| Thailand | 9.4 (8.2-10.7) | 8.3 (7.3-9.3) | 8.8 (8.0-9.6) |
| Timor-Leste | 16.0 (14.1-18.0) | 12.8 (11.2-14.5) | 14.4 (13.2-15.7) |
| *Pooled estimates* | *9.5 (7.0-12.1)* | *10.0 (7.5-12.5)* | *9.8 (7.4-12.3)* |
|  |  |  |  |
| **Western Pacific Region** | | |  |
| Brunei Darussalam | 10.2 (8.5-12.1) | 15.3 (13.4-17.3) | 12.7 (11.4-14.0) |
| Cambodia | 4.7 (3.7-5.8) | 6.5 (5.4-7.7) | 5.5 (4.8-6.3) |
| Cook Islands | 5.9 (3.6-9.0) | 10.0 (7.0-13.7) | 8.1 (6.2-10.5) |
| Fiji | 11.8 (10.2-13.5) | 17.2 (15.4-19.0) | 14.6 (13.4-15.9) |
| French Polynesia | 9.2 (7.7-10.8) | 11.4 (9.9-13.0) | 10.3 (9.2-11.4) |
| Kiribati | 4.9 (3.3-6.8) | 5.5 (4.0-7.2) | 5.2 (4.1-6.5) |
| Laos | 3.1 (2.3-4.1) | 4.7 (3.8-5.8) | 3.9 (3.3-4.6) |
| Malaysia | 6.5 (6.0-6.9) | 9.3 (8.8-9.8) | 7.9 (7.6-8.2) |
| Mongolia | 9.2 (8.1-10.4) | 14.5 (13.2-15.9) | 11.9 (11.1-12.9) |
| Philippines | 12.1 (11.1-13.2) | 19.9 (18.7-21.1) | 16.1 (15.3-16.9) |
| Samoa | 7.7 (5.7-10.1) | 8.9 (7.3-10.8) | 8.4 (7.1-9.8) |
| Solomon Islands | 12.6 (10.1-15.5) | 14.1 (11.4-17.2) | 13.4 (11.5-15.4) |
| Tonga | 11.7 (10.0-13.5) | 15.3 (13.6-17.1) | 13.5 (12.3-14.8) |
| Tuvalu | 9.9 (7.1-13.4) | 8.3 (5.9-11.3) | 9.1 (7.2-11.2) |
| Vanuatu | 9.4 (7.4-11.6) | 9.7 (8.0-11.6) | 9.5 (8.2-10.9) |
| Wallis and Futuna | 12.7 (9.8-16.0) | 16.4 (13.3-19.8) | 14.7 (12.6-17.0) |
| *Pooled estimates* | *8.8 (7.2-10.3)* | *11.6 (9.3-13.9)* | *10.3 (8.4-12.2)* |
|  |  |  |  |
| ***Overall estimate†*** | ***9.7 (8.8-10.6)*** | ***14.4 (13.1-15.8)*** | ***12.1 (11.1-13.2)*** |
| *Country-specific sampling weights were used to yield country representative estimates. | | | |
| †Random-effect meta-analysis was used to calculate the pooled estimates. | | | |

| **Supplementary Table 3: Country-specific, pooled-regional, and pooled-overall prevalence of anxiety among school-going adolescents, by sex and overall** | | | |
| --- | --- | --- | --- |
| **Country** | **Prevalence (95% CI)*** | | |
|  | **Boys** | **Girls** | **Total** |
| **African Region** |  |  |  |
| Benin | 21.3 (19.1-23.6) | 19.5 (17.2-22.0) | 20.9 (19.3-22.5) |
| Ghana | 13.5 (12.0-15.1) | 14.8 (13.1-16.7) | 14.1 (13.0-15.4) |
| Liberia | 17.6 (15.4-19.9) | 21.0 (18.5-23.7) | 19.2 (17.5-21.0) |
| Mauritania | 10.8 (8.8-13.0) | 10.5 (8.6-12.7) | 10.6 (9.2-12.1) |
| Mauritius | 6.5 (5.2-8.0) | 10.4 (8.9-12.1) | 8.7 (7.6-9.8) |
| Mozambique | 7.9 (6.2-10.0) | 11.7 (9.4-14.2) | 9.7 (8.3-11.2) |
| Namibia | 13.7 (12.2-15.3) | 15.7 (14.2-17.3) | 14.8 (13.7-15.9) |
| Seychelles | 7.7 (6.2-9.5) | 13.5 (11.6-15.5) | 10.7 (9.4-12.0) |
| Tanzania | 5.2 (4.2-6.4) | 5.5 (4.5-6.7) | 5.4 (4.6-6.2) |
| *Pooled estimates* | *11.5 (8.2-14.8)* | *13.6 (10.2-16.9)* | *12.6 (9.4-15.9)* |
|  |  |  |  |
| **Eastern Mediterranean Region** |  |  |  |
| Afghanistan | 18.7 (16.2-21.5) | 30.7 (28.0-33.5) | 24.0 (22.1-25.9) |
| Bahrain | 10.2 (9.2-11.3) | 21.5 (20.1-22.9) | 15.9 (15.0-16.8) |
| Iraq | 8.5 (6.9-10.4) | 17.6 (15.1-20.4) | 12.4 (11.0-14.0) |
| Kuwait | 14.8 (13.0-16.8) | 26.5 (24.4-28.7) | 20.9 (19.4-22.3) |
| Lebanon | 9.2 (8.0-10.5) | 16.9 (15.6-18.3) | 13.4 (12.5-14.3) |
| Morocco | 12.7 (11.6-14.0) | 19.8 (18.3-21.3) | 16.1 (15.1-17.0) |
| Palestine | 11.6 (11.0-12.2) | 19.9 (19.3-20.6) | 15.9 (15.5-16.4) |
| United Arab Emirates | 10.0 (8.5-11.7) | 19.6 (17.8-21.5) | 15.0 (13.8-16.3) |
| Yemen | 13.3 (11.3-15.5) | 19.0 (16.7-21.4) | 15.8 (14.3-17.4) |
| *Pooled estimates* | *11.9 (10.5-13.3)* | *21.2 (19.1-23.2)* | *16.5 (15.0-18.0)* |
|  |  |  |  |
| **Region of the Americas** |  |  |  |
| Anguilla | 5.6 (3.4-8.5) | 11.7 (8.7-15.3) | 8.7 (6.8-11.0) |
| Antigua and Barbuda | 11.5 (8.9-14.5) | 16.5 (13.6-19.7) | 13.9 (12.0-16.1) |
| Bahamas | 8.9 (6.6-11.6) | 16.8 (14.1-19.9) | 13.1 (11.3-15.1) |
| Belize | 8.1 (6.4-10.1) | 14.5 (12.4-16.8) | 11.5 (10.1-13.0) |
| Bolivia | 4.9 (3.9-6.1) | 8.6 (7.2-10.0) | 6.7 (5.8-7.6) |
| British Virgin Islands | 4.2 (2.8-6.1) | 14.4 (12.1-16.9) | 9.6 (8.2-11.2) |
| Costa Rica | 3.2 (2.3-4.3) | 6.2 (5.0-7.7) | 4.7 (3.9-5.6) |
| Curacao | 7.3 (5.8-9.0) | 14.7 (12.9-16.6) | 11.2 (10.0-12.5) |
| Dominican Republic | 7.2 (5.1-9.7) | 13.1 (10.7-15.8) | 10.3 (8.7-12.1) |
| El Salvador | 5.1 (3.8-6.7) | 8.9 (7.0-11.0) | 6.9 (5.8-8.2) |
| Guatemala | 4.2 (3.3-5.2) | 8.3 (7.2-9.7) | 6.2 (5.4-7.0) |
| Honduras | 4.5 (3.1-6.2) | 6.6 (5.0-8.5) | 5.6 (4.5-6.8) |
| Jamaica | 8.4 (6.4-10.8) | 16.6 (14.2-19.3) | 12.7 (11.1-14.5) |
| Paraguay | 6.7 (5.4-8.1) | 11.6 (10.0-13.3) | 9.2 (8.2-10.3) |
| Peru | 6.0 (4.8-7.4) | 11.1 (9.5-12.9) | 8.6 (7.6-9.7) |
| Saint Kitts and Nevis | 6.8 (5.0-9.0) | 10.5 (8.6-12.7) | 8.7 (7.3-10.2) |
| Saint Lucia | 10.2 (8.2-12.5) | 16.6 (14.3-19.2) | 13.7 (12.1-15.4) |
| Suriname | 7.9 (6.2-9.8) | 15.0 (12.9-17.4) | 11.6 (10.2-13.1) |
| Trinidad and Tobago | 10.5 (9.1-12.2) | 16.5 (14.9-18.3) | 13.7 (12.5-14.9) |
| Uruguay | 3.0 (2.2-3.9) | 7.9 (6.7-9.3) | 5.6 (4.9-6.5) |
| *Pooled estimates* | *6.6 (5.5-7.6)* | *12.2 (10.6-13.8)* | *9.5 (8.2-10.9)* |
|  |  |  |  |
| **South-East Asia Region** |  |  |  |
| Bangladesh | 3.6 (2.6-4.9) | 4.7 (3.7-5.8) | 4.0 (3.3-4.8) |
| Indonesia | 4.5 (4.0-5.2) | 4.1 (3.6-4.6) | 4.3 (3.9-4.7) |
| Maldives | 11.7 (9.9-13.6) | 18.0 (16.2-19.8) | 14.9 (13.6-16.2) |
| Nepal | 4.2 (3.5-5.0) | 3.8 (3.2-4.5) | 4.0 (3.5-4.5) |
| Sri Lanka | 3.9 (2.9-5.0) | 5.0 (4.0-6.1) | 4.4 (3.7-5.2) |
| Thailand | 7.3 (6.2-8.5) | 8.2 (7.3-9.2) | 7.8 (7.1-8.5) |
| Timor-Leste | 13.0 (11.3-14.8) | 10.8 (9.4-12.4) | 11.9 (10.8-13.1) |
| *Pooled estimates* | *6.7 (4.8-8.7)* | *7.7 (5.2-10.2)* | *7.3 (5.1-9.5)* |
|  |  |  |  |
| **Western Pacific Region** |  |  |  |
| Brunei Darussalam | 7.8 (6.3-9.5) | 13.0 (11.2-14.9) | 10.4 (9.2-11.6) |
| Cambodia | 5.3 (4.3-6.5) | 6.2 (5.1-7.3) | 5.7 (5.0-6.5) |
| Cook Islands | 9.3 (6.3-13.0) | 19.9 (15.8-24.6) | 14.9 (12.3-17.8) |
| Fiji | 10.2 (8.7-11.8) | 13.8 (12.2-15.5) | 12.1 (11.0-13.3) |
| French Polynesia | 7.9 (6.5-9.4) | 15.5 (13.8-17.4) | 11.7 (10.6-12.9) |
| Kiribati | 8.8 (6.7-11.3) | 8.6 (6.8-10.7) | 8.7 (7.3-10.3) |
| Laos | 3.9 (3.0-5.0) | 5.8 (4.8-6.9) | 4.8 (4.1-5.6) |
| Malaysia | 4.5 (4.1-4.9) | 5.7 (5.3-6.1) | 5.1 (4.8-5.4) |
| Mongolia | 5.5 (4.6-6.5) | 5.7 (4.8-6.6) | 5.6 (5.0-6.3) |
| Philippines | 9.2 (8.3-10.2) | 12.5 (11.6-13.6) | 10.9 (10.2-11.6) |
| Samoa | 6.6 (4.8-8.8) | 8.1 (6.6-9.9) | 7.4 (6.2-8.7) |
| Solomon Islands | 9.4 (7.2-12.0) | 14.1 (11.4-17.2) | 11.6 (9.8-13.5) |
| Tonga | 13.9 (12.0-15.8) | 14.7 (13.0-16.4) | 14.3 (13.0-15.6) |
| Tuvalu | 7.3 (4.9-10.4) | 3.8 (2.2-6.1) | 5.4 (4.0-7.2) |
| Vanuatu | 5.5 (4.0-7.3) | 7.4 (5.9-9.1) | 6.5 (5.4-7.7) |
| Wallis and Futuna | 13.5 (10.6-16.9) | 18.0 (14.9-21.5) | 15.8 (13.6-18.2) |
| *Pooled estimates* | *7.9 (6.5-9.2)* | *10.6 (8.6-12.5)* | *9.3 (7.7-10.9)* |
|  |  |  |  |
| ***Overall estimate†*** | ***8.5 (7.6-9.4)*** | ***12.8 (11.4-14.3)*** | ***10.7 (9.6-11.9)*** |
| *Country-specific sampling weights were used to yield country representative estimates. | | | |
| †Random-effect meta-analysis was used to calculate the pooled estimates. | | | |

| **Supplementary Table 4: Country-specific, pooled-regional, and pooled-overall prevalence of suicide ideation among school-going adolescents, by sex and overall** | | | |
| --- | --- | --- | --- |
| **Country** | **Prevalence (95% CI)*** | | |
|  | **Boys** | **Girls** | **Total** |
| **African Region** | |  |  |
| Benin | 12.0 (10.3-13.8) | 17.9 (15.7-20.3) | 13.6 (12.3-15.0) |
| Ghana | 17.2 (15.5-19.0) | 21.3 (19.3-23.5) | 19.2 (17.9-20.5) |
| Liberia | 22.1 (19.7-24.6) | 24.9 (22.2-27.8) | 23.5 (21.6-25.3) |
| Mauritania | 16.3 (13.8-18.9) | 15.8 (13.5-18.3) | 16.0 (14.3-17.8) |
| Mauritius | 11.7 (10.0-13.6) | 19.3 (17.4-21.4) | 15.8 (14.5-17.3) |
| Mozambique | 14.5 (12.2-17.1) | 18.7 (15.9-21.7) | 16.5 (14.7-18.4) |
| Namibia | 18.1 (16.4-19.9) | 19.8 (18.1-21.5) | 19.0 (17.8-20.2) |
| Seychelles | 15.3 (13.2-17.7) | 26.0 (23.6-28.6) | 20.8 (19.2-22.6) |
| Tanzania | 13.6 (12.0-15.4) | 12.1 (10.6-13.7) | 12.8 (11.7-14.0) |
| *Pooled estimates* | *15.6 (13.5-17.6)* | *19.5 (16.5-22.5)* | *17.4 (15.2-19.7)* |
|  |  |  |  |
| **Eastern Mediterranean Region** | | | |
| Afghanistan | 16.5 (14.1-19.2) | 15.2 (13.2-17.4) | 15.9 (14.3-17.6) |
| Bahrain | 12.7 (11.6-13.8) | 16.9 (15.6-18.2) | 14.8 (13.9-15.6) |
| Iraq | 17.1 (14.9-19.6) | 16.5 (14.0-19.2) | 16.9 (15.2-18.6) |
| Kuwait | 14.0 (12.2-15.9) | 18.0 (16.1-19.9) | 16.1 (14.8-17.4) |
| Lebanon | 12.1 (10.7-13.5) | 13.7 (12.5-15.0) | 13.0 (12.1-13.9) |
| Morocco | 12.9 (11.7-14.1) | 17.6 (16.2-19.1) | 15.1 (14.2-16.1) |
| Palestine | 19.2 (18.5-19.9) | 18.9 (18.3-19.6) | 19.1 (18.6-19.5) |
| United Arab Emirates | 11.8 (10.1-13.5) | 14.9 (13.3-16.6) | 13.4 (12.2-14.6) |
| Yemen | 17.6 (15.3-20.0) | 13.6 (11.6-15.7) | 15.7 (14.2-17.3) |
| *Pooled estimates* | *14.8 (12.5-17.2)* | *16.2 (14.6-17.7)* | *15.5 (13.8-17.3)* |
|  |  |  |  |
| **Region of the Americas** | | |  |
| Anguilla | 14.3 (10.8-18.5) | 31.1 (26.6-36.0) | 23.0 (20.0-26.2) |
| Antigua and Barbuda | 13.2 (10.4-16.4) | 21.9 (18.6-25.3) | 17.4 (15.2-19.7) |
| Bahamas | 13.0 (10.3-16.1) | 22.2 (19.1-25.5) | 17.9 (15.8-20.2) |
| Belize | 9.4 (7.6-11.5) | 17.7 (15.5-20.2) | 13.8 (12.3-15.4) |
| Bolivia | 12.8 (11.2-14.4) | 22.7 (20.7-24.8) | 17.6 (16.3-19.0) |
| British Virgin Islands | 10.3 (8.1-12.9) | 18.1 (15.6-20.8) | 14.5 (12.8-16.4) |
| Costa Rica | 7.7 (6.3-9.3) | 13.6 (11.8-15.6) | 10.6 (9.4-11.9) |
| Curacao | 5.8 (4.5-7.4) | 15.6 (13.8-17.6) | 10.9 (9.7-12.2) |
| Dominican Republic | 9.2 (6.9-11.9) | 23.5 (20.5-26.8) | 16.8 (14.8-19.0) |
| El Salvador | 9.3 (7.6-11.4) | 17.5 (15.0-20.3) | 13.4 (11.8-15.0) |
| Guatemala | 11.9 (10.5-13.5) | 22.3 (20.5-24.3) | 16.9 (15.7-18.2) |
| Honduras | 12.8 (10.6-15.4) | 24.0 (21.2-27.0) | 18.8 (17.0-20.8) |
| Jamaica | 15.9 (13.2-18.9) | 32.5 (29.3-35.7) | 24.6 (22.4-26.8) |
| Paraguay | 8.4 (7.0-10.0) | 17.5 (15.7-19.6) | 13.1 (11.9-14.4) |
| Peru | 12.0 (10.3-13.8) | 27.5 (25.2-29.9) | 19.7 (18.2-21.2) |
| Saint Kitts and Nevis | 12.6 (10.2-15.4) | 19.0 (16.5-21.7) | 15.8 (14.0-17.7) |
| Saint Lucia | 13.9 (11.5-16.5) | 31.2 (28.3-34.3) | 23.1 (21.1-25.1) |
| Suriname | 9.9 (8.0-11.9) | 22.0 (19.5-24.7) | 16.1 (14.5-17.8) |
| Trinidad and Tobago | 16.6 (14.8-18.5) | 27.7 (25.7-29.7) | 22.4 (21.0-23.8) |
| Uruguay | 7.1 (5.8-8.5) | 16.2 (14.5-18.0) | 12.0 (10.9-13.1) |
| *Pooled estimates* | *11.2 (9.8-12.5)* | *22.1 (19.8-24.4)* | *16.9 (15.1-18.6)* |
|  |  |  |  |
| **South-East Asia Region** | | |  |
| Bangladesh | 4.4 (3.3-5.8) | 5.8 (4.7-7.0) | 4.9 (4.1-5.8) |
| Indonesia | 3.9 (3.4-4.5) | 5.8 (5.2-6.4) | 4.9 (4.5-5.3) |
| Maldives | 10.2 (8.6-12.0) | 13.3 (11.8-15.0) | 11.8 (10.7-13.0) |
| Nepal | 12.7 (11.5-14.0) | 12.7 (11.5-13.9) | 12.7 (11.9-13.6) |
| Sri Lanka | 9.0 (7.5-10.7) | 8.9 (7.6-10.3) | 8.9 (7.9-10.0) |
| Thailand | 11.0 (9.7-12.4) | 11.1 (10.1-12.3) | 11.1 (10.2-12.0) |
| Timor-Leste | 9.2 (7.8-10.8) | 7.8 (6.5-9.2) | 8.5 (7.6-9.5) |
| *Pooled estimates* | *8.6 (5.6-11.6)* | *9.3 (6.9-11.7)* | *9.0 (6.3-11.6)* |
|  |  |  |  |
| **Western Pacific Region** | | |  |
| Brunei Darussalam | 7.4 (6.0-9.0) | 10.9 (9.2-12.7) | 9.1 (8.0-10.3) |
| Cambodia | 5.4 (4.3-6.5) | 7.0 (5.9-8.2) | 6.1 (5.4-6.9) |
| Cook Islands | 14.2 (10.6-18.5) | 15.8 (12.1-20.2) | 15.0 (12.4-18.0) |
| Fiji | 9.4 (8.0-11.0) | 14.2 (12.6-15.9) | 11.9 (10.8-13.1) |
| French Polynesia | 8.3 (6.9-9.9) | 19.3 (17.4-21.3) | 13.9 (12.7-15.2) |
| Kiribati | 33.0 (29.4-36.8) | 35.2 (32.0-38.6) | 34.2 (31.8-36.7) |
| Laos | 2.2 (1.5-3.0) | 3.4 (2.7-4.3) | 2.7 (2.2-3.3) |
| Malaysia | 6.3 (5.9-6.8) | 9.0 (8.5-9.6) | 7.7 (7.4-8.0) |
| Mongolia | 17.6 (16.0-19.1) | 28.2 (26.6-30.0) | 23.1 (22.0-24.3) |
| Philippines | 8.6 (7.7-9.5) | 13.0 (12.0-14.0) | 10.8 (10.2-11.5) |
| Samoa | 22.3 (19.1-25.7) | 21.5 (19.1-24.0) | 21.9 (19.9-23.9) |
| Solomon Islands | 27.1 (23.6-30.8) | 25.3 (21.8-29.0) | 26.3 (23.8-28.9) |
| Tonga | 12.2 (10.5-14.1) | 10.7 (9.3-12.3) | 11.5 (10.3-12.6) |
| Tuvalu | 9.9 (7.1-13.4) | 4.7 (3.0-7.1) | 7.1 (5.5-9.1) |
| Vanuatu | 17.9 (15.3-20.7) | 11.3 (9.5-13.4) | 14.5 (12.9-16.1) |
| Wallis and Futuna | 17.6 (14.3-21.3) | 29.6 (25.7-33.6) | 23.8 (21.2-26.6) |
| *Pooled estimates* | *13.4 (10.8-16.0)* | *16.0 (12.7-19.4)* | *14.9 (11.9-17.8)* |
|  |  |  |  |
| ***Overall estimate†*** | ***12.7 (11.4-14.0)*** | ***17.8 (16.1-19.4)*** | ***15.3 (13.9-16.8)*** |
| *Country-specific sampling weights were used to yield country representative estimates. | | | |
| †Random-effect meta-analysis was used to calculate the pooled estimates. | | | |

| **Supplementary Table 5: Country-specific, pooled-regional, and pooled-overall prevalence of suicide planning among school-going adolescents, by sex and overall** | | | |
| --- | --- | --- | --- |
| **Country** | **Prevalence (95% CI)*** | | |
|  | **Boys** | **Girls** | **Total** |
| **African Region** | |  |  |
| Benin | 14.5 (12.7-16.5) | 18.1 (15.9-20.5) | 15.5 (14.1-17.0) |
| Ghana | 20.4 (18.6-22.3) | 25.5 (23.4-27.8) | 22.8 (21.4-24.3) |
| Liberia | 35.4 (32.6-38.3) | 34.1 (31.1-37.2) | 34.8 (32.7-36.9) |
| Mauritania | 15.4 (13.1-18.0) | 13.8 (11.7-16.2) | 14.7 (13.1-16.4) |
| Mauritius | 10.7 (9.1-12.5) | 17.1 (15.3-19.1) | 14.2 (12.9-15.6) |
| Mozambique | 18.2 (15.7-21.0) | 19.1 (16.3-22.1) | 18.7 (16.8-20.7) |
| Namibia | 25.1 (23.2-27.1) | 25.0 (23.2-26.9) | 25.0 (23.7-26.4) |
| Seychelles | 16.8 (14.5-19.2) | 24.9 (22.5-27.4) | 20.9 (19.3-22.7) |
| Tanzania | 8.2 (6.9-9.7) | 8.6 (7.3-10.0) | 8.4 (7.5-9.4) |
| *Pooled estimates* | *18.3 (13.3-23.3)* | *20.7 (15.4-25.9)* | *19.4 (14.4-24.4)* |
|  |  |  |  |
| **Eastern Mediterranean Region** | | | |
| Afghanistan | 13.7 (11.5-16.2) | 14.4 (12.4-16.6) | 14.1 (12.6-15.7) |
| Bahrain | 11.8 (10.7-12.9) | 15.5 (14.2-16.7) | 13.6 (12.8-14.5) |
| Iraq | 15.0 (12.9-17.4) | 17.1 (14.6-19.9) | 15.9 (14.3-17.7) |
| Kuwait | 14.5 (12.7-16.5) | 16.7 (14.9-18.6) | 15.6 (14.3-17.0) |
| Lebanon | 7.4 (6.3-8.6) | 8.9 (7.9-9.9) | 8.2 (7.5-9.0) |
| Morocco | 12.5 (11.3-13.7) | 14.0 (12.8-15.4) | 13.2 (12.3-14.1) |
| Palestine | 16.1 (15.4-16.7) | 16.5 (15.9-17.1) | 16.3 (15.9-16.7) |
| United Arab Emirates | 9.1 (7.7-10.8) | 12.3 (10.8-13.9) | 10.8 (9.7-11.9) |
| Yemen | 14.1 (12.1-16.4) | 13.1 (11.2-15.2) | 13.7 (12.2-15.2) |
| *Pooled estimates* | *12.7 (10.4-14.9)* | *14.2 (12.1-16.3)* | *13.5 (11.3-15.6)* |
|  |  |  |  |
| **Region of the Americas** | | |  |
| Anguilla | 17.0 (13.1-21.4) | 25.8 (21.5-30.4) | 21.4 (18.5-24.5) |
| Antigua and Barbuda | 12.6 (9.9-15.8) | 22.3 (19.1-25.9) | 17.4 (15.3-19.8) |
| Bahamas | 11.1 (8.6-14.0) | 17.9 (15.1-21.0) | 14.7 (12.8-16.8) |
| Belize | 14.2 (12.0-16.6) | 18.0 (15.7-20.5) | 16.2 (14.6-17.9) |
| Bolivia | 11.8 (10.3-13.4) | 21.1 (19.1-23.1) | 16.3 (15.1-17.6) |
| British Virgin Islands | 12.0 (9.6-14.7) | 18.8 (16.2-21.6) | 15.6 (13.8-17.5) |
| Costa Rica | 5.4 (4.2-6.8) | 8.8 (7.3-10.4) | 7.1 (6.1-8.1) |
| Curacao | 5.8 (4.5-7.4) | 11.4 (9.8-13.2) | 8.8 (7.7-9.9) |
| Dominican Republic | 8.1 (5.9-10.7) | 20.8 (17.8-23.9) | 14.7 (12.8-16.8) |
| El Salvador | 8.1 (6.4-10.0) | 14.9 (12.6-17.6) | 11.4 (10.0-13.0) |
| Guatemala | 9.5 (8.2-11.0) | 16.9 (15.3-18.6) | 13.1 (12.0-14.2) |
| Honduras | 11.9 (9.7-14.4) | 23.9 (21.1-26.9) | 18.4 (16.5-20.3) |
| Jamaica | 16.9 (14.2-20.0) | 30.1 (27.0-33.3) | 23.8 (21.6-26.0) |
| Paraguay | 9.0 (7.6-10.7) | 15.7 (13.9-17.7) | 12.5 (11.3-13.7) |
| Peru | 9.0 (7.5-10.7) | 21.4 (19.3-23.7) | 15.2 (13.9-16.6) |
| Saint Kitts and Nevis | 14.4 (11.8-17.3) | 16.4 (14.1-19.0) | 15.4 (13.6-17.2) |
| Saint Lucia | 13.0 (10.7-15.5) | 26.5 (23.8-29.4) | 20.2 (18.3-22.1) |
| Suriname | 8.2 (6.5-10.1) | 18.5 (16.1-21.0) | 13.6 (12.1-15.1) |
| Trinidad and Tobago | 16.8 (14.9-18.7) | 25.8 (23.8-27.8) | 21.5 (20.1-22.9) |
| Uruguay | 8.1 (6.7-9.5) | 13.4 (11.8-15.1) | 11.0 (9.9-12.1) |
| *Pooled estimates* | *11.0 (9.5-12.5)* | *19.3 (16.9-21.8)* | *15.3 (13.4-17.3)* |
|  |  |  |  |
| **South-East Asia Region** | | |  |
| Bangladesh | 6.9 (5.5-8.6) | 7.0 (5.9-8.4) | 7.0 (6.0-8.0) |
| Indonesia | 5.3 (4.6-5.9) | 5.5 (5.0-6.1) | 5.4 (5.0-5.8) |
| Maldives | 15.2 (13.3-17.3) | 18.7 (16.9-20.6) | 17.0 (15.7-18.4) |
| Nepal | 12.6 (11.4-13.8) | 13.6 (12.4-14.8) | 13.1 (12.2-14.0) |
| Sri Lanka | 5.9 (4.7-7.2) | 6.0 (4.9-7.2) | 5.9 (5.1-6.8) |
| Thailand | 12.6 (11.2-14.1) | 12.7 (11.5-13.9) | 12.6 (11.8-13.6) |
| Timor-Leste | 9.1 (7.6-10.7) | 7.8 (6.6-9.2) | 8.4 (7.5-9.5) |
| *Pooled estimates* | *9.6 (6.7-12.5)* | *10.1 (6.9-13.3)* | *9.9 (6.9-12.9)* |
|  |  |  |  |
| **Western Pacific Region** | | |  |
| Brunei Darussalam | 4.6 (3.5-5.9) | 8.0 (6.6-9.6) | 6.3 (5.3-7.3) |
| Cambodia | 8.5 (7.2-9.9) | 8.4 (7.2-9.7) | 8.4 (7.5-9.4) |
| Cook Islands | 12.7 (9.2-16.8) | 15.8 (12.1-20.2) | 14.3 (11.7-17.2) |
| Fiji | 11.7 (10.1-13.4) | 15.5 (13.8-17.3) | 13.7 (12.5-14.9) |
| French Polynesia | 11.8 (10.2-13.6) | 20.9 (19.0-22.9) | 16.4 (15.1-17.8) |
| Kiribati | 31.9 (28.3-35.7) | 34.3 (31.1-37.6) | 33.2 (30.8-35.7) |
| Laos | 4.0 (3.1-5.1) | 4.5 (3.6-5.5) | 4.2 (3.6-4.9) |
| Malaysia | 5.2 (4.8-5.6) | 7.1 (6.6-7.5) | 6.2 (5.9-6.5) |
| Mongolia | 12.2 (10.9-13.6) | 16.6 (15.3-18.1) | 14.5 (13.6-15.5) |
| Philippines | 8.9 (8.0-9.8) | 11.6 (10.7-12.6) | 10.3 (9.6-10.9) |
| Samoa | 20.2 (17.1-23.6) | 23.1 (20.6-25.7) | 21.8 (19.8-23.8) |
| Solomon Islands | 26.4 (23.0-30.1) | 24.9 (21.4-28.6) | 25.7 (23.2-28.3) |
| Tonga | 14.8 (12.9-16.8) | 11.7 (10.2-13.3) | 13.2 (12.0-14.5) |
| Tuvalu | 10.7 (7.8-14.2) | 9.5 (6.9-12.6) | 10.0 (8.1-12.3) |
| Vanuatu | 23.7 (20.8-26.9) | 17.1 (14.9-19.5) | 20.3 (18.5-22.2) |
| Wallis and Futuna | 25.7 (21.9-29.9) | 31.2 (27.3-35.3) | 28.6 (25.9-31.5) |
| *Pooled estimates* | *14.3 (11.6-16.9)* | *16.1 (13.1-19.0)* | *15.3 (12.5-18.1)* |
|  |  |  |  |
| ***Overall estimate†*** | ***13.0 (11.7-14.3)*** | ***16.8 (15.3-18.4)*** | ***15.0 (13.6-16.4)*** |
| *Country-specific sampling weights were used to yield country representative estimates. | | | |
| †Random-effect meta-analysis was used to calculate the pooled estimates. | | | |

| **Supplementary Table 6: Country-specific, pooled-regional, and pooled-overall prevalence of suicide attempt among school-going adolescents, by sex and overall** | | | |
| --- | --- | --- | --- |
| **Country** | **Prevalence (95% CI)*** | | |
|  | **Boys** | **Girls** | **Total** |
| **African Region** | |  |  |
| Benin | 16.0 (14.0-18.0) | 13.6 (11.7-15.8) | 15.3 (13.9-16.8) |
| Ghana | 23.5 (21.6-25.5) | 26.4 (24.3-28.7) | 24.9 (23.5-26.4) |
| Liberia | 31.1 (28.4-33.9) | 31.7 (28.8-34.7) | 31.4 (29.4-33.4) |
| Mauritania | 15.7 (13.3-18.3) | 14.5 (12.3-16.9) | 15.1 (13.5-16.8) |
| Mauritius | 9.2 (7.7-10.9) | 13.8 (12.1-15.7) | 11.7 (10.6-13.0) |
| Mozambique | 15.5 (13.1-18.1) | 17.8 (15.1-20.7) | 16.5 (14.7-18.5) |
| Namibia | 26.1 (24.2-28.1) | 23.8 (22.0-25.6) | 24.9 (23.6-26.2) |
| Seychelles | 16.1 (13.9-18.5) | 19.1 (16.9-21.4) | 17.6 (16.1-19.3) |
| Tanzania | 8.1 (6.8-9.5) | 9.6 (8.2-11.0) | 8.8 (7.9-9.8) |
| *Pooled estimates* | *17.9 (12.8-23.0)* | *18.9 (14.3-23.4)* | *18.5 (13.7-23.2)* |
|  |  |  |  |
| **Eastern Mediterranean Region** | | | |
| Afghanistan | 10.9 (8.9-13.1) | 12.3 (10.4-14.3) | 11.5 (10.1-12.9) |
| Bahrain | 11.3 (10.2-12.4) | 12.8 (11.7-14.0) | 12.0 (11.3-12.8) |
| Iraq | 13.3 (11.3-15.5) | 16.4 (13.9-19.1) | 14.6 (13.0-16.3) |
| Kuwait | 10.8 (9.2-12.6) | 15.4 (13.7-17.2) | 13.2 (12.0-14.5) |
| Lebanon | 7.8 (6.7-9.0) | 9.1 (8.1-10.1) | 8.5 (7.8-9.3) |
| Morocco | 9.8 (8.8-11.0) | 12.5 (11.3-13.8) | 11.1 (10.3-12.0) |
| Palestine | 18.6 (17.9-19.3) | 18.4 (17.8-19.1) | 18.5 (18.1-19.0) |
| United Arab Emirates | 9.2 (7.8-10.8) | 9.7 (8.3-11.1) | 9.4 (8.4-10.5) |
| Yemen | 12.5 (10.6-14.7) | 8.9 (7.2-10.7) | 10.9 (9.6-12.3) |
| *Pooled estimates* | *11.6 (8.6-14.6)* | *12.8 (10.0-15.6)* | *12.2 (9.3-15.0)* |
|  |  |  |  |
| **Region of the Americas** | | |  |
| Anguilla | 11.7 (8.5-15.6) | 17.6 (14.0-21.7) | 14.7 (12.2-17.5) |
| Antigua and Barbuda | 9.2 (6.9-12.0) | 16.0 (13.2-19.1) | 12.5 (10.6-14.6) |
| Bahamas | 10.0 (7.6-12.8) | 14.0 (11.5-16.9) | 12.1 (10.4-14.1) |
| Belize | 9.7 (7.9-11.9) | 13.3 (11.3-15.5) | 11.6 (10.3-13.2) |
| Bolivia | 15.7 (14.0-17.6) | 23.5 (21.4-25.6) | 19.5 (18.2-20.9) |
| British Virgin Islands | 7.4 (5.5-9.7) | 13.9 (11.6-16.4) | 10.8 (9.3-12.5) |
| Costa Rica | 5.6 (4.4-7.1) | 9.9 (8.4-11.7) | 7.7 (6.7-8.8) |
| Curacao | 7.5 (6.0-9.2) | 12.4 (10.8-14.3) | 10.1 (8.9-11.3) |
| Dominican Republic | 6.8 (4.8-9.2) | 17.8 (15.1-20.8) | 12.6 (10.8-14.5) |
| El Salvador | 7.2 (5.6-9.1) | 16.3 (13.8-19.0) | 11.7 (10.2-13.3) |
| Guatemala | 10.2 (8.9-11.7) | 19.0 (17.2-20.8) | 14.4 (13.3-15.6) |
| Honduras | 10.9 (8.8-13.3) | 21.9 (19.1-24.8) | 16.8 (15.0-18.7) |
| Jamaica | 12.3 (9.9-15.0) | 19.3 (16.7-22.1) | 15.9 (14.1-17.9) |
| Paraguay | 7.0 (5.7-8.5) | 12.3 (10.7-14.1) | 9.7 (8.7-10.9) |
| Peru | 11.7 (10.0-13.5) | 22.0 (19.9-24.3) | 16.8 (15.4-18.3) |
| Saint Kitts and Nevis | 9.8 (7.7-12.3) | 10.7 (8.8-12.9) | 10.3 (8.8-11.9) |
| Saint Lucia | 10.2 (8.2-12.5) | 17.7 (15.3-20.3) | 14.2 (12.6-15.9) |
| Suriname | 5.9 (4.5-7.6) | 13.2 (11.2-15.4) | 9.7 (8.4-11.1) |
| Trinidad and Tobago | 8.4 (7.1-9.9) | 15.4 (13.8-17.1) | 12.1 (11.0-13.2) |
| Uruguay | 7.4 (6.1-8.8) | 11.1 (9.7-12.7) | 9.4 (8.5-10.5) |
| *Pooled estimates* | *9.1 (8.0-10.3)* | *15.8 (14.0-17.6)* | *12.6 (11.2-14.0)* |
|  |  |  |  |
| **South-East Asia Region** | | |  |
| Bangladesh | 5.7 (4.4-7.2) | 5.8 (4.7-7.0) | 5.7 (4.9-6.6) |
| Indonesia | 3.1 (2.6-3.7) | 3.1 (2.7-3.6) | 3.1 (2.8-3.5) |
| Maldives | 11.5 (9.8-13.4) | 9.0 (7.7-10.4) | 10.2 (9.2-11.4) |
| Nepal | 8.4 (7.4-9.5) | 8.8 (7.8-9.8) | 8.6 (7.9-9.4) |
| Sri Lanka | 5.8 (4.6-7.2) | 6.0 (4.9-7.2) | 5.9 (5.1-6.8) |
| Thailand | 7.9 (6.8-9.2) | 10.0 (8.9-11.1) | 9.1 (8.3-9.9) |
| Timor-Leste | 8.4 (7.0-9.9) | 6.7 (5.6-8.0) | 7.5 (6.6-8.5) |
| *Pooled estimates* | *7.2 (4.9-9.5)* | *7.0 (4.7-9.3)* | *7.2 (4.9-9.5)* |
|  |  |  |  |
| **Western Pacific Region** | | |  |
| Brunei Darussalam | 4.3 (3.2-5.7) | 6.5 (5.3-8.0) | 5.5 (4.6-6.4) |
| Cambodia | 4.9 (3.9-6.0) | 6.6 (5.6-7.9) | 5.7 (5.0-6.5) |
| Cook Islands | 12.3 (9.0-16.4) | 13.2 (9.8-17.3) | 12.8 (10.3-15.6) |
| Fiji | 8.1 (6.7-9.5) | 9.4 (8.1-10.9) | 8.7 (7.8-9.8) |
| French Polynesia | 5.4 (4.3-6.7) | 13.0 (11.4-14.7) | 9.3 (8.3-10.4) |
| Kiribati | 29.9 (26.3-33.6) | 31.1 (28.0-34.3) | 30.5 (28.1-32.9) |
| Laos | 4.0 (3.1-5.1) | 5.6 (4.7-6.8) | 4.8 (4.1-5.5) |
| Malaysia | 5.4 (5.0-5.8) | 6.7 (6.3-7.2) | 6.0 (5.7-6.3) |
| Mongolia | 7.4 (6.4-8.6) | 11.2 (10.0-12.4) | 9.4 (8.6-10.2) |
| Philippines | 11.5 (10.5-12.6) | 16.0 (15.0-17.2) | 13.8 (13.1-14.6) |
| Samoa | 18.6 (15.6-21.9) | 18.4 (16.1-20.8) | 18.4 (16.6-20.4) |
| Solomon Islands | 31.4 (27.7-35.2) | 33.3 (29.5-37.3) | 32.2 (29.6-35.0) |
| Tonga | 15.5 (13.6-17.6) | 12.6 (11.1-14.3) | 14.0 (12.8-15.3) |
| Tuvalu | 10.2 (7.3-13.7) | 3.6 (2.1-5.8) | 6.7 (5.0-8.6) |
| Vanuatu | 25.6 (22.6-28.8) | 17.5 (15.3-19.9) | 21.4 (19.6-23.3) |
| Wallis and Futuna | 11.4 (8.7-14.6) | 15.1 (12.1-18.4) | 13.2 (11.2-15.5) |
| *Pooled estimates* | *12.5 (10.1-14.9)* | *13.5 (10.9-16.1)* | *13.1 (10.7-15.6)* |
|  |  |  |  |
| ***Overall estimate†*** | ***11.5 (10.2-12.8)*** | ***14.2 (12.7-15.7)*** | ***12.9 (11.6-14.3)*** |
| *Country-specific sampling weights were used to yield country representative estimates. | | | |
| †Random-effect meta-analysis was used to calculate the pooled estimates. | | | |

| **Supplementary Table 7: Country-specific, pooled-regional, and pooled-overall prevalence of inadequate fruit intake among school-going adolescents, by sex and overall** | | | |
| --- | --- | --- | --- |
| **Country** | **Prevalence (95% CI)*** | | |
|  | **Boys** | **Girls** | **Total** |
| **African Region** |  |  |  |
| Benin | 37.2 (34.6-39.9) | 34.8 (32.1-37.7) | 36.5 (34.6-38.5) |
| Ghana | 38.8 (36.5-41.0) | 37.7 (35.3-40.2) | 38.3 (36.6-39.9) |
| Liberia | 40.2 (37.3-43.1) | 37.0 (33.9-40.1) | 38.7 (36.6-40.8) |
| Mauritania | 48.1 (44.7-51.5) | 39.1 (36.0-42.3) | 44.1 (41.8-46.4) |
| Mauritius | 41.4 (38.7-44.1) | 39.9 (37.4-42.4) | 40.6 (38.7-42.4) |
| Mozambique | 31.8 (28.7-35.1) | 33.3 (29.9-36.9) | 32.5 (30.2-34.9) |
| Namibia | 43.9 (41.7-46.1) | 44.8 (42.7-46.9) | 44.4 (42.8-45.9) |
| Seychelles | 23.5 (21.0-26.2) | 23.2 (20.9-25.7) | 23.4 (21.7-25.2) |
| Tanzania | 33.3 (31.0-35.7) | 27.7 (25.6-29.9) | 30.4 (28.9-32.0) |
| *Pooled estimates* | *37.6 (33.0-42.1)* | *35.3 (30.5-40.1)* | *36.5 (31.9-41.1)* |
|  |  |  |  |
| **Eastern Mediterranean Region** |  |  |  |
| Afghanistan | 49.8 (46.4-53.2) | 33.4 (30.7-36.3) | 42.5 (40.4-44.7) |
| Bahrain | 44.2 (42.6-45.9) | 52.8 (51.1-54.5) | 48.5 (47.3-49.7) |
| Iraq | 32.2 (29.4-35.1) | 24.9 (21.9-28.0) | 29.0 (27.0-31.2) |
| Kuwait | 41.6 (39.0-44.3) | 44.1 (41.7-46.6) | 42.9 (41.2-44.7) |
| Lebanon | 22.7 (20.9-24.5) | 25.8 (24.3-27.4) | 24.4 (23.2-25.6) |
| Morocco | 28.8 (27.2-30.5) | 25.1 (23.5-26.7) | 27.0 (25.9-28.2) |
| Palestine | 28.7 (27.9-29.5) | 28.3 (27.6-29.1) | 28.5 (28.0-29.1) |
| United Arab Emirates | 42.2 (39.6-44.8) | 48.8 (46.4-51.1) | 45.7 (43.9-47.4) |
| Yemen | 45.4 (42.4-48.5) | 35.4 (32.6-38.3) | 40.9 (38.8-43.0) |
| *Pooled estimates* | *37.2 (31.5-43.0)* | *35.4 (28.5-42.3)* | *36.6 (30.4-42.8)* |
|  |  |  |  |
| **Region of the Americas** |  |  |  |
| Anguilla | 36.0 (30.9-41.3) | 40.1 (35.2-45.1) | 38.1 (34.6-41.8) |
| Antigua and Barbuda | 34.3 (30.2-38.5) | 31.6 (28.0-35.5) | 33.0 (30.3-35.9) |
| Bahamas | 42.9 (38.8-47.2) | 47.7 (43.9-51.5) | 45.6 (42.8-48.4) |
| Belize | 26.0 (23.2-29.0) | 29.5 (26.7-32.4) | 27.8 (25.8-29.9) |
| Bolivia | 29.7 (27.5-31.9) | 28.2 (26.0-30.5) | 28.9 (27.4-30.5) |
| British Virgin Islands | 42.4 (38.6-46.3) | 41.3 (38.0-44.7) | 41.8 (39.3-44.3) |
| Costa Rica | 38.6 (35.9-41.4) | 40.9 (38.3-43.6) | 39.8 (37.9-41.7) |
| Curacao | 41.7 (38.8-44.7) | 38.5 (35.9-41.0) | 40.0 (38.1-42.0) |
| Dominican Republic | 38.9 (34.8-43.1) | 39.1 (35.5-42.8) | 39.0 (36.3-41.8) |
| El Salvador | 30.1 (27.2-33.2) | 22.8 (20.0-25.9) | 26.6 (24.6-28.8) |
| Guatemala | 25.3 (23.3-27.3) | 21.1 (19.3-23.0) | 23.3 (21.9-24.7) |
| Honduras | 33.0 (29.7-36.5) | 32.7 (29.6-36.0) | 32.8 (30.5-35.1) |
| Jamaica | 36.7 (33.1-40.5) | 42.6 (39.3-46.1) | 39.8 (37.3-42.3) |
| Paraguay | 29.1 (26.7-31.6) | 27.6 (25.3-29.9) | 28.3 (26.6-30.0) |
| Peru | 28.1 (25.7-30.6) | 26.8 (24.5-29.2) | 27.5 (25.8-29.2) |
| Saint Kitts and Nevis | 41.5 (37.8-45.4) | 42.7 (39.4-46.0) | 42.1 (39.7-44.6) |
| Saint Lucia | 31.1 (27.9-34.5) | 32.7 (29.7-35.7) | 31.9 (29.7-34.1) |
| Suriname | 27.9 (25.1-30.9) | 28.3 (25.6-31.2) | 28.1 (26.1-30.1) |
| Trinidad and Tobago | 49.3 (46.8-51.8) | 52.3 (50.0-54.6) | 50.9 (49.2-52.6) |
| Uruguay | 27.9 (25.7-30.3) | 30.2 (28.1-32.4) | 29.2 (27.7-30.8) |
| *Pooled estimates* | *34.5 (31.3-37.6)* | *34.8 (30.8-38.8)* | *34.7 (31.2-38.2)* |
|  |  |  |  |
| **South-East Asia Region** |  |  |  |
| Bangladesh | 50.2 (47.2-53.2) | 49.5 (47.1-51.9) | 49.9 (48.0-51.8) |
| Indonesia | 37.6 (36.2-38.9) | 34.9 (33.7-36.1) | 36.2 (35.3-37.1) |
| Maldives | 58.9 (56.1-61.7) | 67.0 (64.8-69.2) | 63.0 (61.3-64.8) |
| Nepal | 54.4 (52.5-56.3) | 51.2 (49.4-53.0) | 52.8 (51.5-54.0) |
| Sri Lanka | 36.6 (34.0-39.2) | 32.2 (30.0-34.5) | 34.3 (32.7-36.0) |
| Thailand | 29.0 (27.1-31.0) | 25.5 (24.0-27.1) | 27.1 (25.9-28.3) |
| Timor-Leste | 53.9 (51.3-56.5) | 54.2 (51.8-56.6) | 54.0 (52.3-55.8) |
| *Pooled estimates* | *45.8 (37.4-54.2)* | *44.9 (34.4-55.5)* | *45.3 (35.9-54.7)* |
|  |  |  |  |
| **Western Pacific Region** |  |  |  |
| Brunei Darussalam | 46.0 (43.1-48.9) | 45.0 (42.3-47.8) | 45.5 (43.6-47.5) |
| Cambodia | 51.0 (48.6-53.4) | 44.4 (42.2-46.7) | 47.9 (46.3-49.5) |
| Cook Islands | 29.0 (24.1-34.3) | 32.6 (27.6-37.8) | 31.0 (27.5-34.6) |
| Fiji | 39.4 (37.0-41.9) | 33.3 (31.0-35.5) | 36.2 (34.5-37.8) |
| French Polynesia | 29.8 (27.4-32.2) | 30.0 (27.8-32.3) | 29.9 (28.3-31.6) |
| Kiribati | 45.8 (41.8-49.7) | 45.1 (41.7-48.6) | 45.4 (42.8-48.0) |
| Laos | 39.9 (37.5-42.4) | 34.6 (32.5-36.8) | 37.5 (35.9-39.1) |
| Malaysia | 33.4 (32.6-34.2) | 30.8 (30.0-31.6) | 32.1 (31.5-32.7) |
| Mongolia | 58.4 (56.4-60.4) | 61.8 (59.9-63.6) | 60.2 (58.8-61.5) |
| Philippines | 36.9 (35.3-38.4) | 36.1 (34.7-37.5) | 36.5 (35.4-37.5) |
| Samoa | 27.7 (24.2-31.4) | 22.6 (20.1-25.2) | 25.0 (22.9-27.1) |
| Solomon Islands | 34.2 (30.4-38.1) | 30.2 (26.5-34.2) | 32.2 (29.6-35.0) |
| Tonga | 28.5 (26.1-31.0) | 27.9 (25.7-30.1) | 28.2 (26.6-29.8) |
| Tuvalu | 41.3 (36.3-46.4) | 37.6 (33.1-42.3) | 39.3 (36.0-42.7) |
| Vanuatu | 23.6 (20.7-26.7) | 22.6 (20.2-25.2) | 23.2 (21.3-25.1) |
| Wallis and Futuna | 42.1 (37.7-46.7) | 35.7 (31.6-39.9) | 38.8 (35.8-41.9) |
| *Pooled estimates* | *38.0 (33.4-42.5)* | *35.6 (30.6-40.6)* | *36.8 (32.1-41.5)* |
|  |  |  |  |
| ***Overall estimate†*** | ***37.6 (35.3-39.8)*** | ***36.4 (33.8-38.9)*** | ***37.0 (34.7-39.4)*** |
| *Country-specific sampling weights were used to yield country representative estimates. | | | |
| †Random-effect meta-analysis was used to calculate the pooled estimates. | | | |

| **Supplementary Table 8: Country-specific, pooled-regional, and pooled-overall prevalence of inadequate vegetable intake among school-going adolescents, by sex and overall** | | | |
| --- | --- | --- | --- |
| **Country** | **Prevalence (95% CI)*** | | |
|  | **Boys** | **Girls** | **Total** |
| **African Region** | |  |  |
| Benin | 37.6 (35.0-40.2) | 32.3 (29.5-35.1) | 36.2 (34.3-38.1) |
| Ghana | 30.0 (27.9-32.2) | 26.1 (23.9-28.4) | 28.1 (26.6-29.7) |
| Liberia | 32.4 (29.7-35.3) | 29.4 (26.6-32.4) | 31.0 (29.0-33.0) |
| Mauritania | 35.9 (32.7-39.2) | 33.4 (30.4-36.6) | 34.8 (32.6-37.0) |
| Mauritius | 13.1 (11.3-15.1) | 12.8 (11.1-14.5) | 13.0 (11.7-14.3) |
| Mozambique | 33.4 (30.2-36.7) | 30.4 (27.1-33.8) | 32.0 (29.7-34.3) |
| Namibia | 41.7 (39.4-43.9) | 40.8 (38.7-42.9) | 41.2 (39.7-42.7) |
| Seychelles | 23.3 (20.8-26.0) | 25.4 (23.0-27.9) | 24.4 (22.6-26.2) |
| Tanzania | 24.6 (22.6-26.8) | 22.4 (20.5-24.4) | 23.5 (22.1-25.0) |
| *Pooled estimates* | *30.2 (23.7-36.7)* | *28.1 (21.9-34.3)* | *29.3 (23.0-35.7)* |
|  |  |  |  |
| **Eastern Mediterranean Region** | | | |
| Afghanistan | 41.1 (37.8-44.5) | 29.8 (27.1-32.5) | 36.1 (34.0-38.3) |
| Bahrain | 40.4 (38.7-42.0) | 44.6 (42.9-46.3) | 42.5 (41.3-43.7) |
| Iraq | 23.7 (21.1-26.4) | 21.4 (18.7-24.4) | 22.7 (20.8-24.7) |
| Kuwait | 31.0 (28.5-33.5) | 31.8 (29.5-34.1) | 31.4 (29.7-33.1) |
| Lebanon | 25.0 (23.1-26.9) | 26.2 (24.7-27.8) | 25.6 (24.5-26.8) |
| Morocco | 15.5 (14.3-16.9) | 16.6 (15.3-18.1) | 16.0 (15.1-17.0) |
| Palestine | 27.9 (27.1-28.7) | 27.2 (26.5-27.9) | 27.5 (27.0-28.1) |
| United Arab Emirates | 37.1 (34.6-39.7) | 37.0 (34.8-39.3) | 37.1 (35.4-38.8) |
| Yemen | 31.4 (28.6-34.3) | 25.6 (23.0-28.2) | 28.7 (26.8-30.7) |
| *Pooled estimates* | *30.3 (24.7-35.8)* | *28.9 (23.6-34.2)* | *29.7 (24.4-35.1)* |
|  |  |  |  |
| **Region of the Americas** | | |  |
| Anguilla | 29.8 (25.0-35.0) | 31.1 (26.6-36.0) | 30.4 (27.1-33.9) |
| Antigua and Barbuda | 24.5 (20.9-28.4) | 27.9 (24.4-31.6) | 26.2 (23.6-28.8) |
| Bahamas | 50.4 (46.1-54.6) | 48.2 (44.3-52.0) | 49.2 (46.4-52.1) |
| Belize | 25.1 (22.3-28.1) | 25.7 (23.0-28.4) | 25.4 (23.4-27.4) |
| Bolivia | 18.9 (17.0-20.8) | 19.0 (17.1-21.0) | 18.9 (17.6-20.3) |
| British Virgin Islands | 32.0 (28.4-35.7) | 35.4 (32.2-38.7) | 33.8 (31.4-36.2) |
| Costa Rica | 26.1 (23.7-28.6) | 24.2 (21.9-26.6) | 25.2 (23.5-26.9) |
| Curacao | 26.2 (23.6-28.9) | 28.4 (26.1-30.8) | 27.3 (25.6-29.1) |
| Dominican Republic | 36.7 (32.6-40.9) | 33.4 (30.0-37.0) | 34.9 (32.3-37.6) |
| El Salvador | 32.2 (29.2-35.3) | 29.5 (26.4-32.8) | 30.9 (28.7-33.1) |
| Guatemala | 24.6 (22.7-26.6) | 22.0 (20.2-23.9) | 23.4 (22.0-24.7) |
| Honduras | 30.1 (26.9-33.5) | 31.6 (28.5-34.9) | 30.9 (28.7-33.3) |
| Jamaica | 30.0 (26.5-33.6) | 35.3 (32.1-38.7) | 32.8 (30.4-35.2) |
| Paraguay | 15.9 (14.0-18.0) | 14.2 (12.5-16.1) | 15.0 (13.7-16.4) |
| Peru | 23.3 (21.1-25.6) | 25.7 (23.5-28.1) | 24.5 (23.0-26.2) |
| Saint Kitts and Nevis | 30.3 (26.8-33.9) | 35.3 (32.2-38.5) | 32.8 (30.5-35.2) |
| Saint Lucia | 38.5 (35.1-42.0) | 37.7 (34.6-40.8) | 38.1 (35.8-40.4) |
| Suriname | 11.2 (9.3-13.4) | 12.1 (10.2-14.3) | 11.7 (10.3-13.2) |
| Trinidad and Tobago | 33.0 (30.7-35.4) | 31.2 (29.1-33.3) | 32.0 (30.5-33.6) |
| Uruguay | 26.2 (24.0-28.5) | 23.6 (21.6-25.6) | 24.8 (23.3-26.3) |
| *Pooled estimates* | *28.1 (24.6-31.7)* | *28.5 (24.9-32.1)* | *28.4 (24.8-31.9)* |
|  |  |  |  |
| **South-East Asia Region** | | |  |
| Bangladesh | 25.9 (23.4-28.6) | 23.6 (21.5-25.7) | 25.1 (23.5-26.8) |
| Indonesia | 19.4 (18.3-20.6) | 15.9 (15.0-16.9) | 17.6 (16.9-18.4) |
| Maldives | 61.7 (58.9-64.4) | 65.2 (63.0-67.5) | 63.5 (61.8-65.2) |
| Nepal | 39.7 (37.9-41.5) | 36.3 (34.7-38.0) | 38.0 (36.8-39.2) |
| Sri Lanka | 12.8 (11.1-14.7) | 6.4 (5.3-7.6) | 9.5 (8.5-10.6) |
| Thailand | 17.6 (16.0-19.3) | 12.0 (10.9-13.2) | 14.5 (13.6-15.5) |
| Timor-Leste | 40.4 (37.8-43.0) | 40.3 (37.9-42.7) | 40.3 (38.6-42.1) |
| *Pooled estimates* | *31.1 (20.2-41.9)* | *28.5 (16.2-40.8)* | *29.8 (18.2-41.4)* |
|  |  |  |  |
| **Western Pacific Region** | | |  |
| Brunei Darussalam | 35.5 (32.7-38.3) | 33.8 (31.2-36.4) | 34.7 (32.8-36.6) |
| Cambodia | 24.7 (22.7-26.8) | 22.0 (20.1-23.9) | 23.4 (22.0-24.8) |
| Cook Islands | 22.5 (18.1-27.5) | 30.5 (25.7-35.7) | 26.6 (23.3-30.1) |
| Fiji | 15.5 (13.7-17.4) | 13.4 (11.9-15.1) | 14.4 (13.2-15.7) |
| French Polynesia | 24.8 (22.6-27.1) | 23.0 (21.0-25.1) | 23.9 (22.4-25.4) |
| Kiribati | 47.8 (43.9-51.8) | 46.5 (43.1-50.0) | 47.1 (44.5-49.7) |
| Laos | 24.2 (22.1-26.3) | 18.8 (17.1-20.6) | 21.7 (20.3-23.1) |
| Malaysia | 19.7 (19.0-20.4) | 18.8 (18.1-19.5) | 19.2 (18.7-19.7) |
| Mongolia | 23.5 (21.8-25.3) | 25.2 (23.6-26.8) | 24.4 (23.2-25.6) |
| Philippines | 20.7 (19.4-22.0) | 19.6 (18.4-20.8) | 20.1 (19.3-21.0) |
| Samoa | 23.9 (20.6-27.4) | 20.5 (18.2-23.1) | 22.1 (20.2-24.1) |
| Solomon Islands | 22.5 (19.2-26.0) | 16.0 (13.1-19.2) | 19.5 (17.3-21.8) |
| Tonga | 24.4 (22.1-26.8) | 22.7 (20.7-24.7) | 23.5 (22.0-25.1) |
| Tuvalu | 37.3 (32.5-42.4) | 34.7 (30.3-39.3) | 35.9 (32.6-39.3) |
| Vanuatu | 24.6 (21.7-27.8) | 19.7 (17.4-22.2) | 22.1 (20.2-24.0) |
| Wallis and Futuna | 53.1 (48.5-57.6) | 49.3 (45.0-53.6) | 51.1 (48.0-54.2) |
| *Pooled estimates* | *27.5 (24.3-30.8)* | *25.6 (22.4-28.8)* | *26.7 (23.6-29.9)* |
|  |  |  |  |
| ***Overall estimate†*** | ***29.0 (26.7-31.2)*** | ***27.8 (25.3-30.3)*** | ***28.5 (26.1-30.8)*** |
| *Country-specific sampling weights were used to yield country representative estimates. | | | |
| †Random-effect meta-analysis was used to calculate the pooled estimates. | | | |

| **Supplementary Table 9: Country-specific, pooled-regional, and pooled-overall prevalence of daily soft drink consumption among school-going adolescents, by sex and overall** | | | |
| --- | --- | --- | --- |
| **Country** | **Prevalence (95% CI)*** | | |
|  | **Boys** | **Girls** | **Total** |
| **African Region** | |  |  |
| Benin | 32.1 (29.6-34.7) | 42.1 (39.2-45.0) | 34.8 (32.9-36.7) |
| Ghana | 42.4 (40.1-44.7) | 47.9 (45.4-50.4) | 45.0 (43.3-46.7) |
| Liberia | 40.4 (37.5-43.3) | 50.2 (47.0-53.4) | 45.0 (42.8-47.2) |
| Mauritania | 45.1 (41.8-48.6) | 55.2 (51.9-58.4) | 49.7 (47.3-52.0) |
| Mauritius | 40.9 (38.2-43.7) | 39.5 (37.1-42.1) | 40.2 (38.4-42.0) |
| Mozambique | 52.9 (49.5-56.4) | 60.2 (56.5-63.7) | 56.4 (53.9-58.9) |
| Namibia | 46.2 (43.9-48.4) | 46.7 (44.6-48.8) | 46.5 (44.9-48.0) |
| Seychelles | 67.4 (64.5-70.3) | 68.9 (66.2-71.5) | 68.1 (66.2-70.1) |
| Tanzania | 39.9 (37.5-42.3) | 49.0 (46.6-51.3) | 44.6 (42.9-46.3) |
| *Pooled estimates* | *45.2 (39.0-51.5)* | *51.0 (45.1-57.0)* | *47.8 (41.7-53.9)* |
|  |  |  |  |
| **Eastern Mediterranean Region** | | | |
| Afghanistan | 35.9 (32.7-39.2) | 36.3 (33.5-39.2) | 36.1 (33.9-38.2) |
| Bahrain | 39.3 (37.6-40.9) | 28.8 (27.3-30.4) | 34.1 (32.9-35.2) |
| Iraq | 56.1 (53.1-59.2) | 49.3 (45.8-52.8) | 53.2 (50.9-55.5) |
| Kuwait | 54.5 (51.8-57.2) | 50.5 (48.1-53.0) | 52.4 (50.6-54.2) |
| Lebanon | 52.2 (50.1-54.4) | 42.2 (40.5-43.9) | 46.8 (45.5-48.2) |
| Morocco | 30.6 (28.9-32.3) | 31.8 (30.1-33.6) | 31.1 (30.0-32.4) |
| Palestine | 56.1 (55.2-57.0) | 52.6 (51.8-53.4) | 54.3 (53.7-54.9) |
| United Arab Emirates | 35.2 (32.7-37.7) | 23.7 (21.7-25.7) | 29.2 (27.6-30.8) |
| Yemen | 39.6 (36.6-42.6) | 31.7 (29.0-34.5) | 36.0 (34.0-38.1) |
| *Pooled estimates* | *44.4 (36.8-51.9)* | *38.5 (30.3-46.7)* | *41.5 (33.8-49.1)* |
|  |  |  |  |
| **Region of the Americas** | | |  |
| Anguilla | 51.5 (46.0-56.9) | 52.0 (47.0-57.1) | 51.6 (48.0-55.3) |
| Antigua and Barbuda | 58.0 (53.7-62.3) | 57.4 (53.4-61.4) | 57.7 (54.8-60.6) |
| Bahamas | 65.9 (61.8-69.9) | 71.8 (68.2-75.1) | 69.2 (66.5-71.7) |
| Belize | 64.2 (61.0-67.4) | 63.5 (60.5-66.5) | 63.9 (61.7-66.0) |
| Bolivia | 63.2 (60.9-65.5) | 63.0 (60.6-65.4) | 63.1 (61.4-64.8) |
| British Virgin Islands | 65.2 (61.4-68.8) | 63.1 (59.8-66.4) | 64.1 (61.7-66.6) |
| Costa Rica | 50.7 (47.9-53.5) | 52.0 (49.3-54.7) | 51.3 (49.4-53.3) |
| Curacao | 60.2 (57.2-63.1) | 60.7 (58.1-63.2) | 60.5 (58.5-62.4) |
| Dominican Republic | 71.6 (67.6-75.3) | 78.6 (75.4-81.5) | 75.2 (72.7-77.6) |
| El Salvador | 65.0 (61.8-68.0) | 68.5 (65.2-71.7) | 66.7 (64.4-68.9) |
| Guatemala | 64.8 (62.6-66.9) | 57.5 (55.3-59.8) | 61.3 (59.7-62.9) |
| Honduras | 73.3 (70.0-76.4) | 74.9 (71.9-77.8) | 74.1 (71.9-76.3) |
| Jamaica | 71.4 (67.8-74.8) | 64.3 (61.0-67.6) | 67.7 (65.3-70.1) |
| Paraguay | 61.8 (59.2-64.4) | 57.3 (54.7-59.8) | 59.5 (57.7-61.3) |
| Peru | 54.5 (51.8-57.2) | 53.0 (50.3-55.6) | 53.7 (51.8-55.6) |
| Saint Kitts and Nevis | 61.9 (58.1-65.6) | 59.6 (56.3-62.8) | 60.7 (58.2-63.1) |
| Saint Lucia | 54.4 (50.9-57.9) | 50.7 (47.5-53.9) | 52.4 (50.1-54.8) |
| Suriname | 79.0 (76.3-81.6) | 78.9 (76.2-81.3) | 78.9 (77.1-80.7) |
| Trinidad and Tobago | 55.5 (53.0-57.9) | 49.2 (46.9-51.5) | 52.2 (50.5-53.8) |
| Uruguay | 72.2 (69.9-74.4) | 69.1 (66.9-71.2) | 70.5 (68.9-72.1) |
| *Pooled estimates* | *63.2 (59.8-66.7)* | *62.3 (58.2-66.3)* | *62.7 (59.1-66.4)* |
|  |  |  |  |
| **South-East Asia Region** | | |  |
| Bangladesh | 46.4 (43.4-49.4) | 46.6 (44.2-49.1) | 46.4 (44.6-48.3) |
| Indonesia | 30.1 (28.8-31.5) | 25.1 (24.0-26.2) | 27.5 (26.7-28.4) |
| Maldives | 37.2 (34.5-40.0) | 28.4 (26.3-30.6) | 32.8 (31.1-34.5) |
| Nepal | 30.2 (28.5-31.9) | 34.0 (32.3-35.7) | 32.1 (30.9-33.3) |
| Sri Lanka | 27.6 (25.3-30.1) | 23.5 (21.5-25.6) | 25.5 (24.0-27.1) |
| Thailand | 57.2 (55.1-59.4) | 52.3 (50.5-54.1) | 54.5 (53.1-55.9) |
| Timor-Leste | 41.3 (38.7-43.9) | 42.2 (39.8-44.6) | 41.7 (40.0-43.5) |
| *Pooled estimates* | *38.6 (30.6-46.6)* | *36.0 (27.5-44.5)* | *37.2 (29.2-45.3)* |
|  |  |  |  |
| **Western Pacific Region** | | |  |
| Brunei Darussalam | 51.9 (49.0-54.8) | 40.4 (37.7-43.1) | 46.2 (44.2-48.2) |
| Cambodia | 40.9 (38.6-43.3) | 42.6 (40.4-44.9) | 41.7 (40.1-43.3) |
| Cook Islands | 56.2 (50.6-61.7) | 53.7 (48.2-59.1) | 55.0 (51.2-58.9) |
| Fiji | 62.9 (60.5-65.3) | 61.5 (59.2-63.8) | 62.1 (60.5-63.8) |
| French Polynesia | 45.0 (42.4-47.7) | 46.7 (44.3-49.2) | 45.9 (44.1-47.7) |
| Kiribati | 18.9 (15.9-22.1) | 24.3 (21.4-27.3) | 21.7 (19.7-23.9) |
| Laos | 48.5 (46.0-51.0) | 51.9 (49.6-54.1) | 50.1 (48.4-51.7) |
| Malaysia | 31.4 (30.6-32.3) | 26.4 (25.6-27.2) | 28.9 (28.3-29.5) |
| Mongolia | 37.6 (35.7-39.6) | 29.5 (27.8-31.2) | 33.4 (32.1-34.7) |
| Philippines | 38.8 (37.2-40.4) | 35.7 (34.3-37.2) | 37.2 (36.2-38.3) |
| Samoa | 60.4 (56.5-64.3) | 65.2 (62.3-68.0) | 62.9 (60.6-65.2) |
| Solomon Islands | 43.7 (39.7-47.7) | 42.6 (38.6-46.7) | 43.2 (40.3-46.0) |
| Tonga | 56.2 (53.5-58.9) | 63.2 (60.9-65.5) | 59.7 (58.0-61.5) |
| Tuvalu | 46.2 (41.1-51.4) | 55.6 (50.9-60.3) | 51.3 (47.8-54.7) |
| Vanuatu | 39.9 (36.5-43.4) | 37.4 (34.6-40.4) | 38.6 (36.4-40.8) |
| Wallis and Futuna | 52.7 (48.1-57.2) | 61.5 (57.3-65.7) | 57.3 (54.2-60.3) |
| *Pooled estimates* | *45.6 (40.0-51.3)* | *46.1 (39.0-53.2)* | *45.9 (39.7-52.2)* |
|  |  |  |  |
| ***Overall estimate†*** | ***50.3 (46.9-53.8)*** | ***49.8 (46.0-53.6)*** | ***50.0 (46.5-53.6)*** |
| *Country-specific sampling weights were used to yield country representative estimates. | | | |
| †Random-effect meta-analysis was used to calculate the pooled estimates. | | | |

| **Supplementary Table 10: Country-specific, pooled-regional, and pooled-overall prevalence of weekly fast food consumption among school-going adolescents, by sex and overall** | | | |
| --- | --- | --- | --- |
| **Country** | **Prevalence (95% CI)*** | | |
|  | **Boys** | **Girls** | **Total** |
| **African Region** |  |  |  |
| Benin | 38.5 (35.9-41.2) | 38.6 (35.7-41.5) | 38.5 (36.6-40.5) |
| Ghana | 61.6 (59.3-63.9) | 65.6 (63.1-67.9) | 63.6 (61.9-65.2) |
| Liberia | 37.5 (34.6-40.4) | 40.5 (37.4-43.7) | 38.9 (36.8-41.0) |
| Mauritania | 56.4 (53.0-59.7) | 67.3 (64.2-70.3) | 61.3 (59.0-63.6) |
| Mauritius | 58.0 (55.2-60.7) | 56.9 (54.4-59.5) | 57.4 (55.6-59.3) |
| Mozambique | 56.4 (53.0-59.8) | 63.0 (59.4-66.5) | 59.5 (57.0-62.0) |
| Namibia | 51.8 (49.6-54.1) | 45.8 (43.7-48.0) | 48.6 (47.1-50.2) |
| Seychelles | 67.9 (65.0-70.8) | 69.0 (66.3-71.5) | 68.5 (66.5-70.4) |
| Tanzania | 34.1 (31.8-36.5) | 34.4 (32.2-36.7) | 34.2 (32.6-35.9) |
| *Pooled estimates* | *51.3 (43.4-59.3)* | *53.5 (44.4-62.5)* | *52.3 (43.9-60.6)* |
|  |  |  |  |
| **Eastern Mediterranean Region** |  |  |  |
| Afghanistan | 71.1 (68.0-74.1) | 56.2 (53.2-59.1) | 64.5 (62.4-66.6) |
| Bahrain | 73.0 (71.5-74.5) | 75.4 (73.9-76.8) | 74.2 (73.1-75.2) |
| Iraq | 62.1 (59.0-65.0) | 48.5 (45.0-52.0) | 56.3 (54.0-58.5) |
| Kuwait | 78.1 (75.8-80.3) | 72.8 (70.5-74.9) | 75.4 (73.8-76.9) |
| Lebanon | 80.0 (78.3-81.7) | 74.9 (73.3-76.4) | 77.3 (76.1-78.4) |
| Morocco | 66.3 (64.6-68.0) | 60.7 (58.9-62.5) | 63.7 (62.4-64.9) |
| Palestine | 55.6 (54.7-56.5) | 42.6 (41.8-43.4) | 48.8 (48.2-49.4) |
| United Arab Emirates | 75.3 (73.0-77.5) | 72.6 (70.5-74.6) | 73.9 (72.3-75.4) |
| Yemen | 38.4 (35.5-41.4) | 33.6 (30.8-36.5) | 36.3 (34.2-38.3) |
| *Pooled estimates* | *66.7 (58.8-74.6)* | *59.7 (48.3-71.1)* | *63.4 (53.8-72.9)* |
|  |  |  |  |
| **Region of the Americas** |  |  |  |
| Anguilla | 75.1 (70.2-79.6) | 76.0 (71.5-80.2) | 75.6 (72.3-78.7) |
| Antigua and Barbuda | 51.0 (46.6-55.3) | 62.8 (58.8-66.6) | 56.8 (53.9-59.7) |
| Bahamas | 68.5 (64.4-72.3) | 72.8 (69.3-76.1) | 70.8 (68.2-73.3) |
| Belize | 64.0 (60.8-67.1) | 65.9 (62.9-68.7) | 65.0 (62.8-67.1) |
| Bolivia | 57.9 (55.5-60.3) | 57.1 (54.6-59.5) | 57.5 (55.8-59.2) |
| British Virgin Islands | 50.9 (47.0-54.8) | 58.9 (55.5-62.2) | 55.2 (52.7-57.8) |
| Costa Rica | 51.5 (48.7-54.3) | 56.3 (53.6-59.0) | 53.8 (51.9-55.8) |
| Curacao | 66.1 (63.2-68.9) | 71.2 (68.8-73.5) | 68.8 (66.9-70.6) |
| Dominican Republic | 41.3 (37.1-45.5) | 52.9 (49.2-56.6) | 47.4 (44.6-50.2) |
| El Salvador | 54.4 (51.1-57.6) | 58.6 (55.1-62.0) | 56.4 (54.1-58.8) |
| Guatemala | 56.3 (54.0-58.6) | 57.1 (54.8-59.3) | 56.7 (55.1-58.3) |
| Honduras | 45.6 (42.0-49.2) | 47.1 (43.7-50.5) | 46.4 (43.9-48.8) |
| Jamaica | 53.8 (50.0-57.7) | 60.4 (56.9-63.7) | 57.3 (54.7-59.8) |
| Paraguay | 55.4 (52.7-58.1) | 52.2 (49.7-54.8) | 53.8 (51.9-55.6) |
| Peru | 51.7 (49.0-54.4) | 49.0 (46.4-51.6) | 50.4 (48.5-52.3) |
| Saint Kitts and Nevis | 55.2 (51.3-59.0) | 64.0 (60.8-67.2) | 59.6 (57.1-62.0) |
| Saint Lucia | 56.9 (53.4-60.4) | 64.6 (61.5-67.6) | 61.0 (58.6-63.3) |
| Suriname | 63.6 (60.4-66.7) | 61.7 (58.6-64.7) | 62.6 (60.4-64.7) |
| Trinidad and Tobago | 66.2 (63.8-68.5) | 70.8 (68.7-72.9) | 68.6 (67.1-70.2) |
| Uruguay | 43.5 (41.0-46.1) | 46.4 (44.0-48.7) | 45.1 (43.4-46.8) |
| *Pooled estimates* | *56.4 (53.0-59.9)* | *60.3 (56.5-64.0)* | *58.4 (54.9-61.9)* |
|  |  |  |  |
| **South-East Asia Region** |  |  |  |
| Bangladesh | 56.0 (53.1-59.0) | 47.5 (45.1-49.9) | 53.1 (51.2-54.9) |
| Indonesia | 52.2 (50.8-53.6) | 55.9 (54.6-57.2) | 54.1 (53.2-55.1) |
| Maldives | 40.7 (38.0-43.5) | 36.0 (33.8-38.3) | 38.4 (36.6-40.1) |
| Nepal | 73.4 (71.7-75.0) | 76.5 (75.0-78.0) | 75.0 (73.9-76.1) |
| Sri Lanka | 39.3 (36.7-41.9) | 43.2 (40.8-45.6) | 41.3 (39.5-43.0) |
| Thailand | 79.7 (77.9-81.4) | 83.6 (82.3-84.9) | 81.9 (80.8-82.9) |
| Timor-Leste | 61.8 (59.3-64.4) | 66.5 (64.2-68.8) | 64.2 (62.5-65.9) |
| *Pooled estimates* | *57.6 (46.2-69.1)* | *58.5 (45.3-71.7)* | *58.3 (46.1-70.5)* |
|  |  |  |  |
| **Western Pacific Region** |  |  |  |
| Brunei Darussalam | 66.9 (64.1-69.6) | 65.1 (62.4-67.6) | 66.0 (64.1-67.9) |
| Cambodia | 21.3 (19.4-23.3) | 21.1 (19.3-23.0) | 21.2 (19.9-22.5) |
| Cook Islands | 67.9 (62.5-73.0) | 68.9 (63.7-73.8) | 68.4 (64.7-71.9) |
| Fiji | 62.3 (59.9-64.8) | 60.5 (58.1-62.8) | 61.3 (59.6-63.0) |
| French Polynesia | 72.0 (69.6-74.3) | 71.7 (69.4-73.8) | 71.8 (70.2-73.4) |
| Kiribati | 45.3 (41.4-49.2) | 40.7 (37.4-44.1) | 42.9 (40.3-45.5) |
| Laos | 42.7 (40.3-45.1) | 39.9 (37.7-42.2) | 41.4 (39.8-43.1) |
| Malaysia | 45.6 (44.7-46.5) | 47.8 (46.9-48.7) | 46.7 (46.1-47.3) |
| Mongolia | 55.7 (53.7-57.7) | 54.4 (52.5-56.3) | 55.0 (53.7-56.4) |
| Philippines | 47.9 (46.3-49.5) | 48.0 (46.5-49.5) | 47.9 (46.9-49.0) |
| Samoa | 64.6 (60.7-68.3) | 69.7 (66.9-72.4) | 67.3 (65.0-69.5) |
| Solomon Islands | 65.0 (61.1-68.8) | 67.0 (63.0-70.8) | 66.0 (63.2-68.7) |
| Tonga | 67.7 (65.1-70.2) | 70.6 (68.4-72.8) | 69.2 (67.5-70.8) |
| Tuvalu | 46.0 (40.9-51.1) | 36.7 (32.2-41.4) | 41.0 (37.6-44.4) |
| Vanuatu | 54.6 (51.1-58.1) | 53.5 (50.5-56.5) | 54.1 (51.8-56.3) |
| Wallis and Futuna | 65.8 (61.3-70.0) | 63.2 (59.0-67.3) | 64.5 (61.5-67.4) |
| *Pooled estimates* | *55.7 (49.0-62.4)* | *54.9 (48.0-61.8)* | *55.3 (48.6-62.0)* |
|  |  |  |  |
| ***Overall estimate†*** | ***57.1 (53.8-60.5)*** | ***57.6 (53.9-61.2)*** | ***57.4 (54.0-60.8)*** |
| *Country-specific sampling weights were used to yield country representative estimates. | | | |
| †Random-effect meta-analysis was used to calculate the pooled estimates. | | | |
